# Genome-wide CRISPR screen identifies genes synthetically lethal with *GRA17*, a nutrient channel encoding gene in *Toxoplasma*

**DOI:** 10.1371/journal.ppat.1011543

**Published:** 2023-07-27

**Authors:** Tatiana C. Paredes-Santos, Mebratu A. Bitew, Christopher Swale, Felipe Rodriguez, Shruthi Krishnamurthy, Yifan Wang, Parag Maru, Lamba Omar Sangaré, Jeroen P. J. Saeij

**Affiliations:** 1 Department of Pathology, Microbiology and Immunology, School of Veterinary Medicine, University of California Davis, Davis, California, United States of America; 2 Team Host-Pathogen Interactions and Immunity to Infection, Institute for Advanced Biosciences (IAB), INSERM U1209, CNRS UMR5309, University Grenoble Alpes, Grenoble, France; University of Wisconsin Medical School, UNITED STATES

## Abstract

*Toxoplasma gondii* is a parasite that replicates within a specialized compartment called the parasitophorous vacuole (PV), which is surrounded by the PV membrane (PVM). To obtain essential nutrients, *Toxoplasma* must transport molecules across the PVM, a process mediated by the secreted parasite proteins GRA17 and GRA23. These proteins form pores in the PVM through which small molecules can diffuse in and out of the PV. *GRA17* and *GRA23* are synthetically lethal, suggesting that at least one pore type is essential for parasite survival. In the ‘nutrient sensitized’ Δ*gra17* strain it is likely that other *Toxoplasma* genes become essential, because they mediate nutrient acquisition from the host or are involved in the trafficking of GRA23 to the PVM. To identify these genes, a genome-wide loss-of-function screen was performed in wild-type and Δ*gra17* parasites, which identified multiple genes that were synthetically sick/lethal with *GRA17*. Several of these genes were involved in the correct localization of GRAs, including GRA17/GRA23, to the PVM. One of the top hits, GRA72, was predicted to form a pore on the PVM, and its deletion led to the formation of enlarged “bubble vacuoles” with reduced PVM small molecule permeability, similar to what was previously observed for Δ*gra17* parasites. Furthermore, Δ*gra72* parasites had reduced *in vitro* growth and virulence in mice. These findings suggest that in the absence of GRA17, other genes become essential, likely because they play a role in the proper localization of GRA23 (and other GRAs) or because they determine host-derived nutrient acquisition at the PVM.

## Author summary

Pathogens that reside in a membrane-bound vacuole inside host cells need to overcome the vacuole membrane barrier to acquire host nutrients. The vacuolar parasite *Toxoplasma gondii* makes pores in the vacuole membrane using two proteins, GRA17 and GRA23, that are required for the diffusion of small host molecules, such as nutrients, across the membrane. GRA17 and GRA23 are exocytosed from the parasite’s dense granule secretory organelles into the vacuolar lumen. However, the exact mechanism that subsequently determines their proper localization into the vacuole membrane is not known. In parasites that are missing the *GRA17* gene (Δ*gra17*), GRA23 becomes a critical factor for parasite survival. We exploited this vulnerability by conducting a screen that determined what parasite genes are essential in Δ*gra17* but not in wild-type parasites. This screen identified multiple *Toxoplasma* genes that encode for proteins that are required for the correct localization of multiple dense granule proteins, including GRA17 and GRA23. Because dense granule proteins can mediate host-parasite interactions, the parasite proteins that are needed for the correct localization of dense granule proteins are critical for parasite fitness. Our screen also identified a dense granule protein with the predicted ability to form pores in the vacuole membrane. These pores are likely to play a role in facilitating the acquisition of host nutrients.

## Introduction

*Toxoplasma gondii* is a leading cause of foodborne mortality worldwide [1]. *Toxoplasma*’s replication niche is the parasitophorous vacuole (PV), which is a subcellular compartment in the host cytosol created when it invades host cells. Like all obligate intracellular parasites, *Toxoplasma* needs to acquire certain nutrients from its host [2], but accessing these nutrients is hindered by the PV membrane (PVM) that encloses the replicating parasites. However, small host nutrients likely can cross the PVM through permeability pores formed by the dense granule proteins (GRAs) GRA17 and GRA23, that allow the diffusion of small molecules up to 1.3–1.9 kDa [3,4]. These pores may also allow the efflux of toxic waste products generated by the parasite. The slow growth and avirulence of Δ*gra17* parasites in mice suggest that certain host-derived nutrients become limiting to parasite growth in the absence of GRA17 PVM-pores. GRA17 and GRA23 function synergistically and are synthetically lethal in *Toxoplasma*, indicating that the parasite needs at least one pore-type to survive [4]. Because classical transporters have not been identified on the PVM [5], *Toxoplasma* has developed additional mechanisms to acquire host nutrients that are not freely diffusible through the GRA17/GRA23 PVM pores. One such mechanism involves intercepting and ‘ingesting’ different types of host vesicles and cytosolic proteins. For instance, *Toxoplasma* can intercept Rab-derived vesicles loaded with sphingolipids [6], lipid droplets containing neutral lipids [7], and endolysosomes filled with cholesterol [8]. By degrading the limiting membrane of these vesicles through the action of lecithin:cholesterol acyltransferase (*Tg*LCAT), which is secreted into the PV lumen, their cargo is released into the PV [6]. In addition, *Toxoplasma* can ingests host cytosolic proteins [9], which eventually end up in *Toxoplasma*’s vacuolar compartment (VAC), a lysosomal-like organelle, where their digestion could provide *Toxoplasma* with amino acids, similar to how *Plasmodium* spp. acquire amino acids from ingested hemoglobin [10]. The recruitment of the host Endosomal Sorting Complex Required for Transport (ESCRT) to the PVM, mediated by GRA14 and GRA64 [11,12], is likely necessary for the ingestion of host cytosolic proteins by promoting the budding and scission of host-derived vesicles into the PV lumen [13]. In Δ*gra2* parasites, the ingestion of host cytosolic proteins, Rab-vesicles, and lipid droplets is significantly reduced [6]. This is likely because the intravacuolar network (IVN) of membranous tubules [14], which is stabilized by the tubulogenic GRA2 protein [15], provides a conduit for the transport of nutrients to *Toxoplasma*. Thus, multiple GRA proteins play important roles in the acquisition of host-derived nutrients.

In order for GRA17 and GRA23 to function as nutrient pores in the PVM, their insertion into other membranes *en route* to the PVM needs to be prevented. Once they are exocytosed from dense granule organelles into the PV lumen, they also need to be transported to and inserted into the PVM. However, all the parasite proteins involved in these processes have not been identified. For transmembrane domain-containing GRA5 and GRA6, the N-terminal sequence between their signal peptide and their transmembrane domain is important for their correct localization to the PVM post secretion, suggesting it contains a sorting element [16,17]. We recently identified GRA45 as an important chaperone-like protein that possibly shields the hydrophobic domain of GRAs in the secretory pathway *en route* to dense granule organelles, preventing their insertion into the ER membrane [18]. Without GRA45, many GRAs, including GRA17/GRA23, fail to properly localize to the PVM, although a small fraction still manages to reach this location. Recently, the With-No-Gly-loop (WNG)1 kinase, which can phosphorylate multiple GRAs, was shown to facilitate their insertion into the PVM post dense granule exocytosis into the PV lumen [19]. WNG1 may mediate the dissociation of hydrophobic GRA cargo from the chaperone by phosphorylating GRAs or phosphorylating chaperones in the PV lumen that bind to GRAs [20].

To investigate the essentiality of GRA23 pores in the PVM and other genes involved in host-derived nutrient acquisition in *Δgra17* parasites, we performed a genome-wide synthetic lethality screen. The results showed that multiple genes had a significantly larger fitness effect when deleted in Δ*gra17* compared to wild-type parasites, and a subset of these were confirmed to be synthetically sick/lethal with *GRA17*. The majority of the identified genes were found to be involved in the correct localization of GRA23, GRA17 and other GRAs. Our screen also identified a dense granule protein (GRA72) that is predicted to be able to form pores in the PVM. The potential involvement of this protein in nutrient acquisition, and its functional relationship with other GRAs, warrants further investigation. Our results highlight the complex molecular interplay that determines *Toxoplasma*’s ability to acquire host nutrients. They also underscore the need for additional research to fully understand how the identified gene products impact the parasite’s fitness and survival strategy.

## Results

### Adaptation of Δ*gra17* parasites

**Fig 1:**
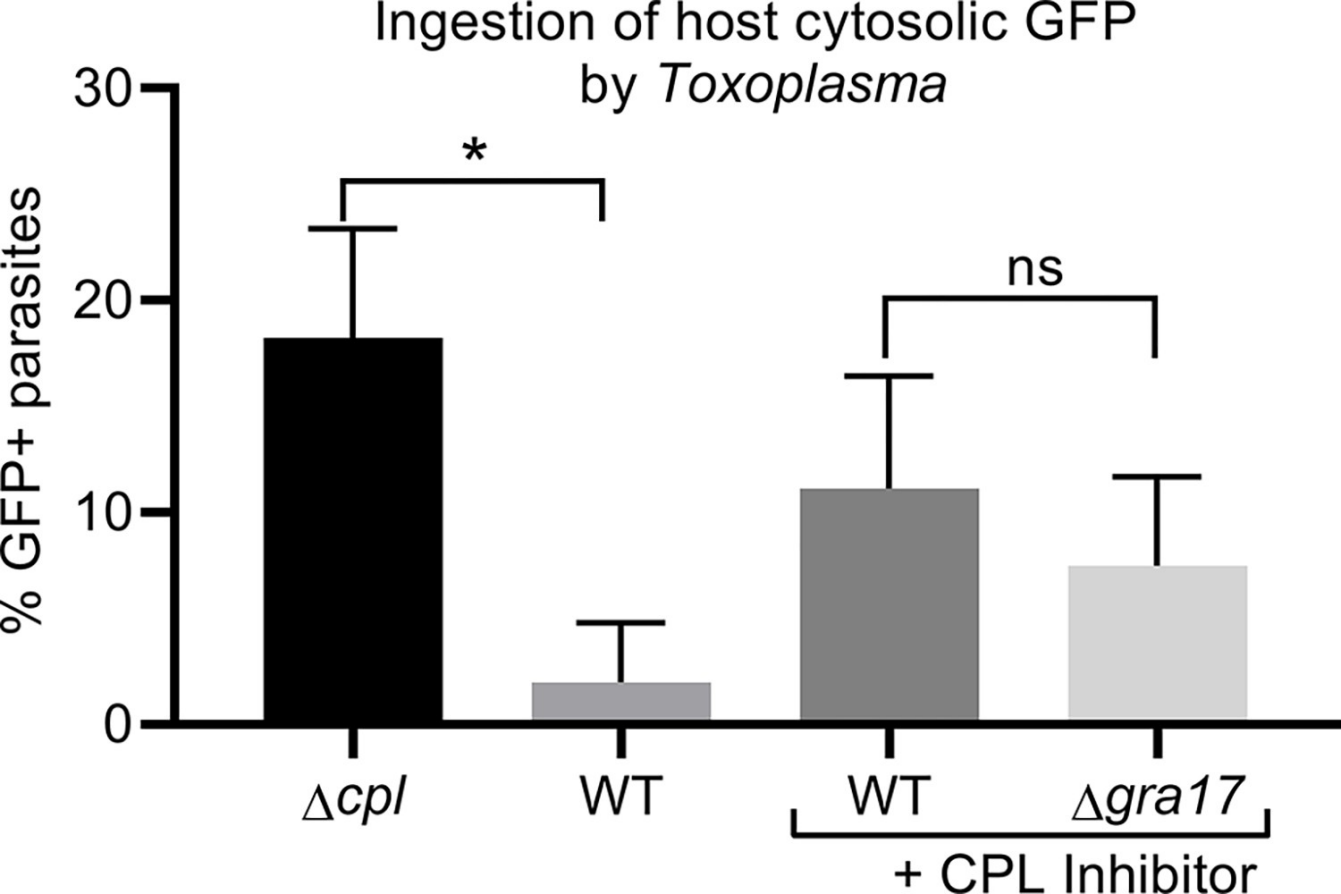
Ingestion of host cytosolic proteins is not upregulated in Δ*gra17* parasites. CHO cells were transfected with a GFP-expressing plasmid and 24 h after transfection cells were infected with indicated parasites treated or not with 1 μM LHVS. 24 h p.i., parasites were harvested and the percentage of parasites containing GFP in the vacuolar compartment (VAC) was determined with a fluorescent microscope. Shown are means±SD from three independent experiments. Significance was assessed by one way ANOVA with Tukey’s multiple comparison test. * Indicates *P*<0.05. ns indicates “not significant”.

We previously observed that when *GRA17* is deleted the Δ*gra17* parasites initially have strongly reduced growth and mostly form grossly enlarged “bubble” vacuoles that have reduced permeability to small molecules [4]. However, after many passages the Δ*gra17* parasites start to grow faster with fewer bubble vacuoles. We hypothesized that other nutrient uptake mechanisms are upregulated in Δ*gra17* parasites to compensate for the loss of GRA17 and the likely resulting decreased influx of small host nutrients. Increased ingestion of host cytoplasmic proteins could potentially compensate for the loss of GRA17. To determine if there is increased uptake of host cytoplasmic proteins in Δ*gra17* parasites we transfected a GFP-expression plasmid into CHO cells, and 20 h later infected them with *Toxoplasma* for 24 h and subsequently quantified the percentage of parasites with a GFP-positive VAC. Ingestion of host cytosolic proteins is only detected in strains where the Cathepsin L (*CPL)* gene is deleted or inhibited [9]. Similar to previously published results [9], we observed that the Δ*cpl* strain and strains treated with the (CPL) inhibitor LHVS have a higher percentage of GFP-positive VAC-containing parasites compared to untreated parasites. However, the uptake of host cytosolic GFP was similar between CPL-treated wild-type and Δ*gra17* parasites (**Fig 1**). Thus, Δ*gra17* parasites do not appear to upregulate the ingestion of host proteins to compensate for the lack of GRA17.

By comparing RNAseq data of wild-type and Δ*gra17* parasites (from a later passage) we previously observed that in the Δ*gra17* strain *GRA23* is upregulated [4]. We further analyzed these RNAseq data previously generated from the following strains: wild-type, Δ*gra17* parasites complemented with *GRA17* but with ~2.5 fold lower expression of *GRA17* compared to wild-type, a GRA17 overexpression strain, and Δ*gra17*, to identify other genes that are differentially regulated in Δ*gra17* parasites. Besides G*RA23*, 81 other genes had at least a 1.5 fold higher expression in Δ*gra17* (and RPKM ≥ 10 in Δ*gra17*) compared to the wild-type and GRA17 overexpression strains (**S1 Table**). Two other genes encoding for GRAs, TGGT1_272460 (*GRA72*) and TGGT1_203600 (*CST2/GRA50*), were 1.8 and 2.7 fold higher expressed in Δ*gra17* parasites, respectively. Four genes encoding for membrane transport proteins that belong to the Major Facilitator Family (MFS) were also upregulated in Δ*gra17* parasites (**S1 Table**). These genes are *TgAT1* (TGGT1_244440), a purine transporter [21], *TgApiAT2* (TGGT1_320020), *TgApiAT5-3*, a tyrosine transporter (TGGT1_257530), and *TgApiAT6-2* (TGGT1_290860)[22]. Thus, the Δ*gra17* strain likely adapts to the reduced influx of host derived nutrients by upregulating GRA23 and by upregulating nutrient transporters on its plasma membrane.

#### Genome-wide loss-of-function screen identifies *Toxoplasma* genes that are synthetically lethal/sick with *GRA17*

**Fig 2:**
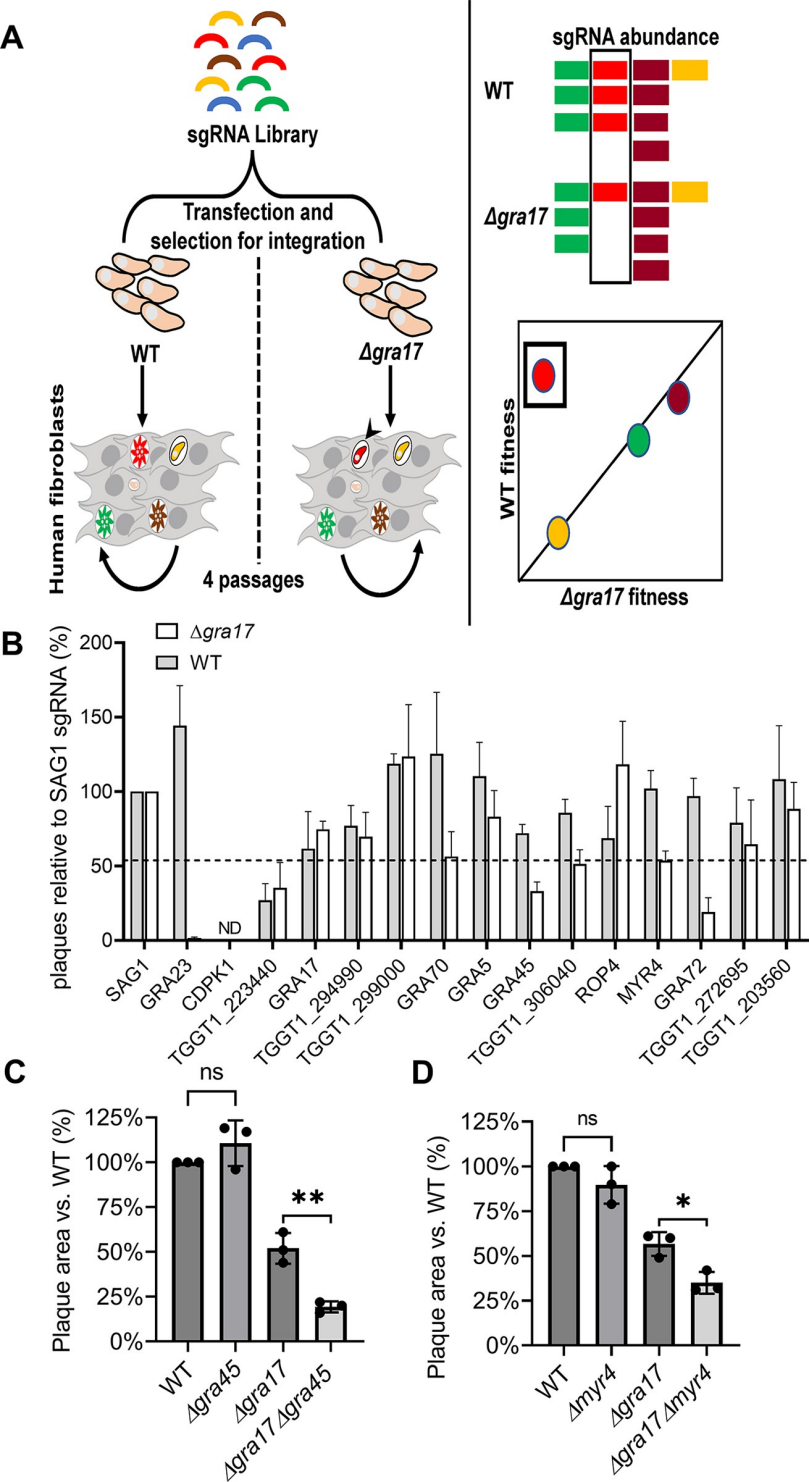
Genome-wide CRISPR screen identifies *Toxoplasma* genes that are synthetically sick/lethal with *GRA17*. **A) CRISPR synthetic lethality screen procedure.** Wild-type (WT) or Δ*gra17* parasites expressing Cas9 were transfected with CRISPR plasmids containing 10 different sgRNAs against each of 8,156 *Toxoplasma* genes. The pool of parasite mutants was passaged in HFFs for four rounds under pyrimethamine selection to select for parasites that integrated the sgRNA-containing plasmid. The abundance of sgRNAs at the 3rd and 4^th^ passage was determined by Illumina sequencing and used to calculate fitness scores to identify genes that have a fitness defect in Δ*gra17* but not in WT parasites. **B) Identification of genes with a larger fitness defect when deleted in Δ*gra17* vs. wild-type parasites.** WT and Δ*gra17* Cas9-expressing parasites were electroporated with two plasmids containing a pyrimethamine resistance cassette and a different sgRNA targeting one of the hits from the synthetic lethality screen (**Table 1**). After electroporation 5,000 parasites were added to 6-well plates containing HFFs and 3 μg/ml (1μM) pyrimethamine and incubated for 8 days after which the number of plaques were counted. Plotted are the percentage of plaques formed in WT or Δ*gra17* parasites after transfection with two sgRNAs targeting the indicated genes relative to the number of plaques after transfection with the SAG1-targeting sgRNAs. Error bars are SD (n = 3 biological replicates). ND = not detected. **C-D) *GRA45* and *MYR4* are synthetically sick with *GRA17***. WT or knockout parasite strains were used to infect HFFs and five days post infection the plaque areas were quantified. Relative parasite growth of knockout parasites was calculated relative to the plaque area of WT parasites (set at 100%). The results are shown as mean ± SD from three independent experiments. Statistical analysis was done with One-way ANOVA followed by Tukey’s multiple comparison test (*p < .05, **p < .01).

We hypothesized that in the absence of the GRA17 nutrient pore, other *Toxoplasma* gene products that mediate: (i) nutrient acquisition from the host at the PVM; or (ii) the trafficking of proteins involved in nutrient acquisition to the PVM after their secretion into the PV lumen, become essential in the ‘nutrient sensitized’ Δ*gra17* strain. We therefore performed a CRISPR/Cas9-mediated genome-wide loss-of-function screen in the ‘nutrient sensitized’ Δ*gra17* and the wild-type strain to identify genes that have a strong fitness defect in Δ*gra17* but not in wild-type parasites (synthetic lethality/sickness) **Fig 2A**). Compared to wild type, Δ*gra17* parasites are less viable, which made it challenging to maintain the complexity of the mutant pool and prevent random loss of mutants. Because of these challenges we were only able to generate one dataset. We therefore set strict filtering criteria and focused on genes that at passage 3 and 4 of the mutant pool: 1) had at least a 16-fold stronger fitness defect in Δ*gra17 vs*. wild-type (phenotype score difference ≤-4); 2) had a phenotype score of ≤-4 in Δ*gra17*; 3) were expressed (RPKM>10) in Δ*gra17* and/or wild-type tachyzoites (as previously determined by RNAseq [4]); and 4) were targeted by sgRNAs that were significantly (P-value <0.05) negatively selected in Δ*gra17 vs*. wild-type; 85 genes fulfilled these criteria (**S1 Table**). *GRA23*, which has no phenotype when deleted in wild-type parasites but is synthetically lethal in Δ*gra17* [4], was in our hit list demonstrating the screen is able to identify a known gene that is synthetically lethal with *GRA17*. At the 4th passage, *GRA23* had a phenotype score of -5.0 in Δ*gra17* and 1.5 in wild-type parasites (**Table 1**). Thus, GRA23 loss-of-function mutants in the Δ*gra17*, but not the wild-type background, rapidly get lost, consistent with *GRA23* being synthetically lethal with *GRA17*. To determine if these 85 genes had any functional enrichment, we performed a pathway enrichment analysis (**S2 Table**). There was a significant enrichment in genes involved in glycerophospholipid metabolism (e.g., TgLCAT, TGGT1_226370 encoding for a 2-acylglycerol O-acyltransferase 1 (DGAT2L1)-like gene, TGGT1_254690/ Active Serine Hydrolase (TgASH1) a depalmitoylating enzyme, and TGGT1_288740/ L-Asparaginase) and glycogen degradation / carbohydrate binding (TGGT1_225490/CDPK2, TGGT1_265450 hexokinase, and TGGT1_314910 encoding for a starch-binding domain containing protein). This could potentially indicate that without GRA17 the parasite needs to rely more on glycogen and lipid degradation for its energy. Thus, besides *GRA23*, multiple other genes appear to have a larger fitness defect when knocked out in Δ*gra17* vs. wild-type parasites.

**Table 1:** Hits from the CRISPR screen that were selected for confirmation. Phenotype scores for the indicated parasite genes in Δ*gra17* vs. WT parasites at the 4th passage are shown. Also included are the P-values (average from 3rd/4th passage), which were calculated using the MAGeCK algorithm. For all data and further details, please refer to **S1 Table**.

Gene ID	Product Description	LOPIT	Δgra17 phenotype	WT phenotype	P-value
TGGT1_294990	hypothetical protein		-6.1	2.9	6.6E-05
TGGT1_299000	hypothetical protein		-5.0	1.8	1.6E-04
TGGT1_249990	hypothetical protein/GRA70	dense granules	-6.3	1.5	2.8E-04
TGGT1_286450	GRA5	dense granules	-5.6	2.6	7.5E-04
TGGT1_297880	GRA23	dense granules	-5.0	1.5	2.4E-03
TGGT1_316250	GRA45	dense granules	-5.0	1.8	5.1E-03
TGGT1_306040	CHY zinc finger protein	nucleus—non-chromatin	-5.8	1.6	6.5E-03
TGGT1_295125	ROP4	rhoptries 1	-5.8	0.5	7.0E-03
TGGT1_211460	MYR4	dense granules	-4.8	2.2	1.3E-02
TGGT1_272460	hypothetical protein/GRA72	dense granules	-5.1	0.3	2.0E-02
TGGT1_272695	hypothetical protein	ER	-4.9	2.7	2.4E-02
TGGT1_203560	hypothetical protein		-4.2	1.9	2.8E-02

### Confirmation of hits from the genome-wide synthetic lethality screen

To confirm hits in our candidate list, we transfected Δ*gra17* or wild-type parasites constitutively expressing Cas9 with two plasmids expressing a different sgRNA against 11 genes from the 85 hits (**Table 1**) and performed plaque assays under conditions that selected for integration of the plasmid into the genome (**Fig 2B**). As a control we used sgRNAs against *GRA23* (synthetically lethal with *GRA17*), the essential gene *CDPK1* [23], and the fitness-conferring gene *TGGT1_223440* (phenotype score -3.62 [24]). sgRNAs against five of the 11 genes (*GRA70*, *GRA45*, *TGGT1_306040*, *MYR4*, and *GRA72*) tested led to fewer plaques in Δ*gra17* parasites compared to the SAG1 control sgRNAs (~2-fold vs. SAG1 sgRNAs) and to ~1.5 fold fewer plaques in Δ*gra17* vs. wild-type parasites. MYR4 and GRA45 were previously shown to be dense granule proteins that are localized to the PV [18,25] while GRA70 and GRA72 are potential dense granule proteins predicted by hyper-LOPIT [26] (ToxoDB.org). Consistent with this, we recently showed that C-terminally 3xHA endogenously tagged versions of GRA70 and GRA72 co-localize with dense granule proteins and are secreted into the PV [27]. TGGT1_306040 is predicted to be a nuclear protein and is annotated as a CHY zinc finger protein in ToxoDB, but it also contains a SANT/Myb domain. While this protein might potentially regulate the expression of other genes that have differential fitness effects in wild-type vs. Δ*gra17* parasites, we did not further investigate this gene. To further determine if the GRA-encoding genes play critical roles in the growth of Δ*gra17* parasites, we tried to obtain knockout clones of these genes in wild-type and Δ*gra17* parasites. We were able to obtain the single knockout clones of *MYR4* and *GRA45* in both wild-type and Δ*gra17* parasites. Although the Δ*gra45* and Δ*myr4* parasites had normal overall growth as determined by plaque assays, the Δ*gra17*Δ*gra45* and Δ*gra17*Δ*myr4* parasites formed significantly smaller plaques compared to Δ*gra17* parasites (**Fig 2C–2D**) indicating that *GRA45* and *MYR4* are synthetically sick with *GRA17*.

For *GRA70* and *GRA72* we were only able to obtain knockout clones in wild-type parasites despite two attempts to obtain knockouts in Δ*gra17* parasites. We also failed to obtain knockout clones when trying to delete *GRA17* in the Δ*gra70* and Δ*gra72* parasites. These data are consistent with *GRA70* and *GRA72* being synthetically lethal with *GRA17* or at least having a very large fitness effect in that background. It is important to note that we have confirmed that the plaques formed when Δ*gra17* was transfected with sgRNAs against *GRA70* or *GRA72* (**Fig 2B**) are from parasites in which these genes are not disrupted but which contain silent small deletions (e.g., 3 base pair deletion). Taken together, we identified four dense granule proteins contributing to the parasite fitness in the ‘nutrient sensitized’ Δ*gra17* background.

### Multiple screen hits are required for the proper localization of GRAs

**Fig 3:**
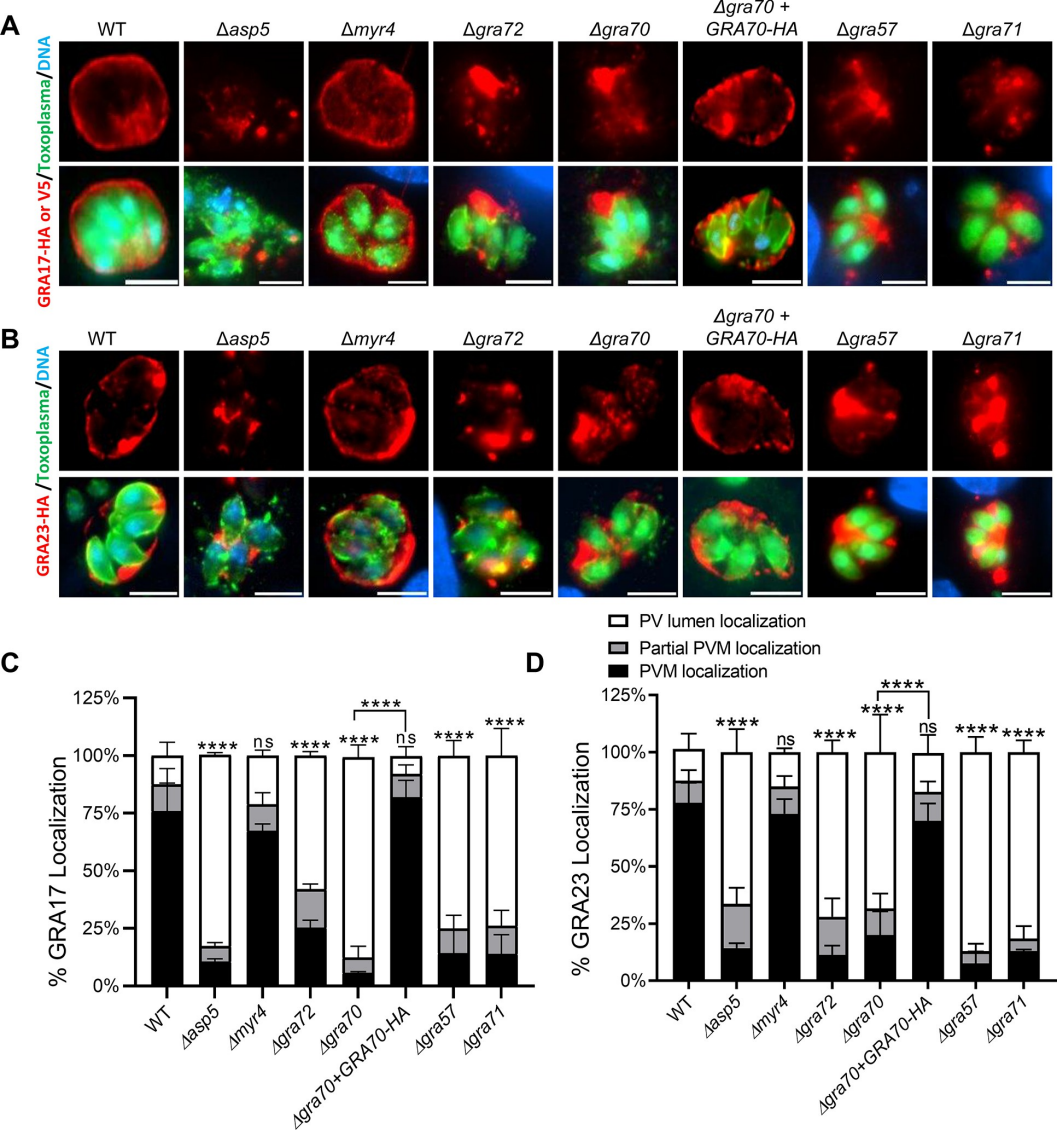
Localization of GRA17 and GRA23 in knockout parasites. HFFs were infected with the indicated parasite strains transiently expressing GRA17-HA, GRA17-V5, or GRA23-HA at an MOI of 0.5 for 24 h. Representative images of **A)** GRA17 and **B)** GRA23 localization in the different strains. Scale bar = 8μm. The percentage of vacuoles with PV lumen, PVM, or partial PVM staining of **C)** GRA17 or **D)** GRA23 was quantified. Statistical significance was determined by Two way-ANOVA with Dunnett’s multiple comparisons test. (****p < .0001, n = 12 for WT, n = 3 for other strains).

We previously showed that GRA45, which was one of our top hits, is a chaperone-like protein important for the correct localization of GRA proteins to the PVM. In Δ*gra45* parasites, GRA23 is mislocalized and mostly retained in the PV lumen [18]. Moreover, we previously observed that Δ*gra17* parasites, when complemented with a GRA17 construct that resulted in ~2.5 fold lower expression of GRA17, did not display any phenotype [4]. The lack of a fitness effect in Δ*gra45* parasites therefore suggests that adequate amounts of GRA17 likely still reach the PVM. Since the correct localization of GRA23 to the PVM is probably more important for survival of Δ*gra17* parasites, this likely explains the synthetic sickness of *GRA45* with *GRA17*. Therefore, a possible explanation for the synthetic lethality/sickness of *MYR4*, *GRA70*, and *GRA72* with *GRA17* is that they play a role in the correct trafficking of GRA23 to the PVM. To examine if GRA17/GRA23 proteins are mislocalized in Δ*myr4*, Δ*gra70*, and Δ*gra72* parasites, we transiently expressed HA-tagged GRA23 and HA-tagged or V5-tagged GRA17 in these parasites and quantified if their localization appeared to be PVM or PV lumen. As a control, we used Δ*asp5* parasites that are known to grossly mislocalize GRA proteins [28–30]. In contrast to the mislocalization of GRA17 and GRA23 in Δ*asp5* parasites, these proteins were not mislocalized in Δ*myr4* parasites. Similar to what we observed in Δ*asp5* parasites, a significantly larger fraction of GRA17 and GRA23 was retained in the PV lumen in Δ*gra72* and Δ*gra70* compared to wild-type parasites (**Fig 3**). Complementation of Δ*gra70* parasites with an HA-tagged copy of GRA70, restored the localization of GRA17/GRA23 (**Fig 3**).

We previously showed that GRA70 can immunoprecipitate TGGT1*_*217680 (GRA57), TGGT1*_*309600 (GRA71), and TGGT1*_*212300 (GRA32) and that these proteins are each other’s 1st or 2nd best BLAST hit [27]. GRA57 was in our list of top hits from the synthetic lethality screen while GRA71 has a phenotype score of -5.5 vs. -0.6 in Δ*gra17* vs. wild-type parasites, respectively, but missed the P-value cut-off for passage 4 (**S1 Table**). In contrast, GRA32 was not in our list of hits. Δ*gra57* and Δ*gra71* parasites also showed significantly more retention of GRA17 and GRA23 proteins in the PV lumen as compared to the parental parasite strain (**Fig 3**).

**Fig 4:**
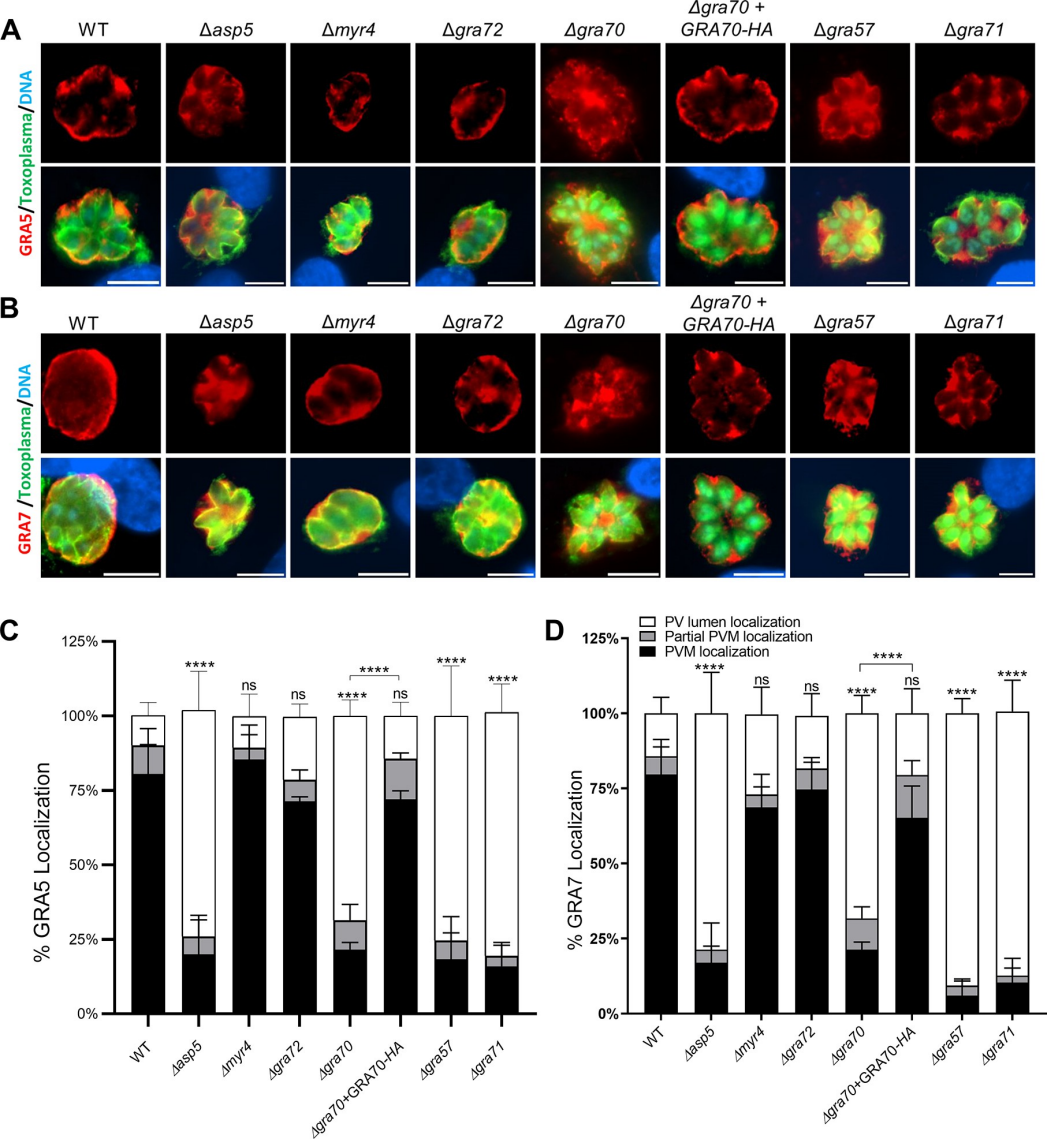
Localization of GRA5 and GRA7 in knockout parasites. HFFs were infected with the indicated parasite strains at an MOI of 0.5 for 24 h. Representative images of **A)** GRA5 and **B)** GRA7 localization in the different strains. Scale bar = 9μm. The percentage of vacuoles with PV lumen, PVM, or partial PVM staining for **C)** GRA5 or **D)** GRA7 was quantified. Statistical significance was determined by Two way-ANOVA with Dunnett’s multiple comparisons test (****p < .0001, n = 3).

To determine if these GRAs are important for the correct localization of other PVM associated proteins, in addition to GRA17 and GRA23, we quantified the proportion of vacuoles where GRA5 and GRA7 appeared localized to either the PV lumen or PVM. In the positive control Δ*asp5* parasites, GRA5 and GRA7 were primarily retained in the PV lumen, while these proteins exhibited normal, wild-type-like PVM localization in Δ*myr4* parasites (**Fig 4**). Contrary to the mislocalization we observed for GRA17 and GRA23, GRA5 and GRA7 displayed normal localization in Δ*gra72* parasites. However, in Δ*gra70* knockout parasites, we observed a significantly greater retention of both GRA5 and GRA7 in the PV lumen, which reverted to wild-type levels in the complemented strain (Δ*gra70+GRA70-HA*). Similarly, in Δ*gra57* and Δ*gra71* parasites, GRA5 and GRA7 were predominantly retained within the PV lumen (**Fig 4**).

Δ*gra72* parasites had similar export of GRA16 and GRA24 as wild-type and complemented parasites suggesting that GRA export beyond the PVM is not affected by deletion of *GRA72* (**S1 Fig**). It was recently published that GRA57 also plays no role in GRA export [31]. Thus, the role of GRA45, GRA57, GRA70, GRA71, and GRA72 in the correct localization of GRA23 could explain their synthetic sickness/lethality with GRA17, although the role of other PVM proteins cannot be excluded.

### Deletion of *GRA72* results in the formation of ‘bubble’ vacuoles with reduced permeability

**Fig 5:**
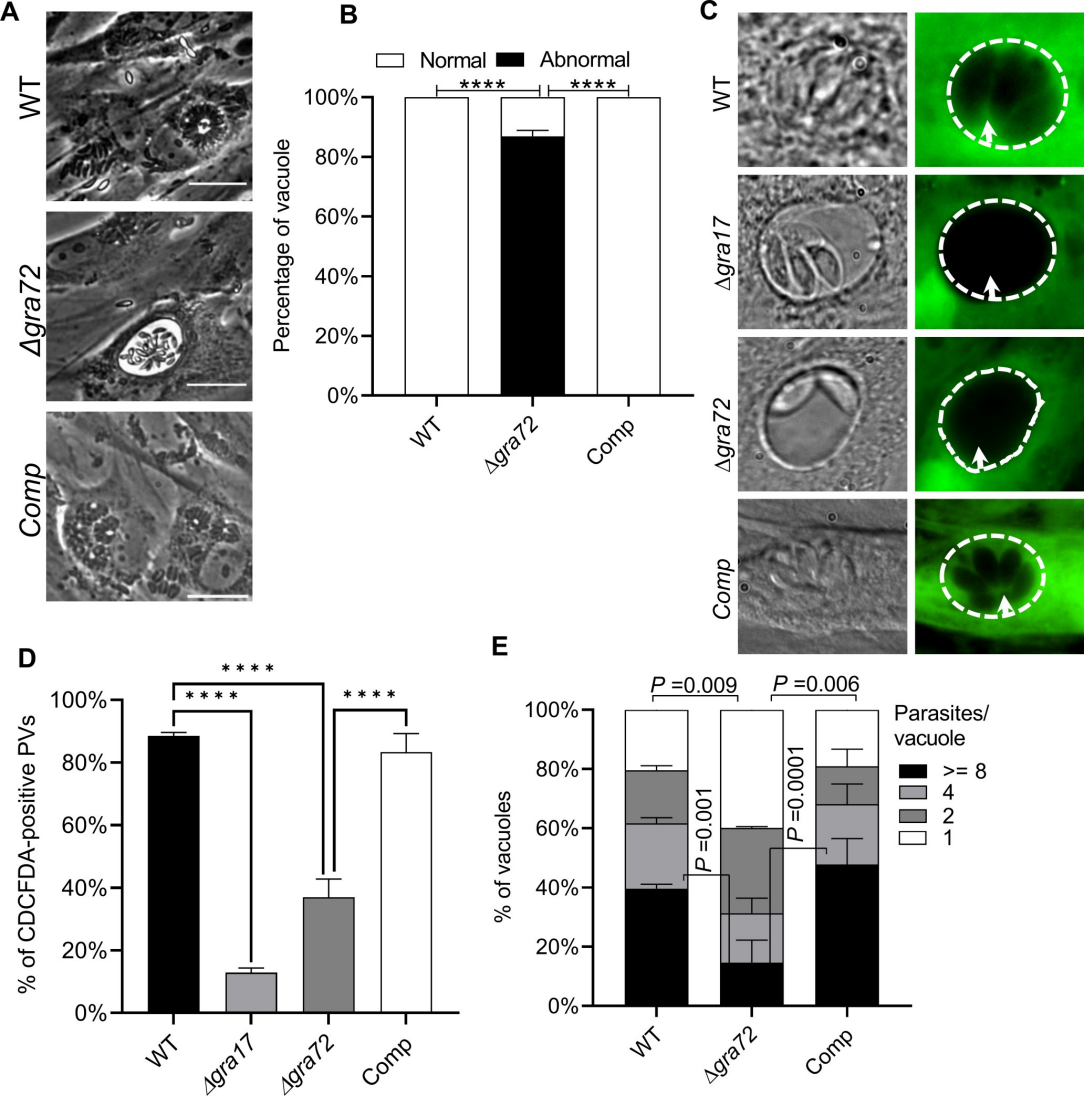
Δ*gra72* parasites form bubble vacuoles with decreased PVM permeability to small molecules. **A)** HFFs were infected with indicated parasite strains at an MOI of 1 for 36 h (WT = wild-type, Comp = Δ*gra72*+GRA72). Shown are representative examples of a bubble (abnormal vacuole) in Δ*gra72* parasites and normal vacuole in WT and complemented parasites. **B)** Vacuoles were quantified as normal or abnormal from at least 20 microscopic fields. Vacuoles with abnormal morphology include bubble vacuoles, enlarged collapsed vacuoles and vacuoles containing opaque tachyzoites. Both normal and abnormal vacuoles were quantified from three different passages (infections). Two way-ANOVA with Dunnett’s multiple comparison test was used to determine statistical significance (****p < .0001, n = 3) **C)** HFFs were infected with indicated parasite strains for 24 h and subsequently pulsed with CDCFDA for 10 minutes after which the dye was washed away and vacuoles were imaged. Shown are representative images from the wild-type and complemented strain showing normal permeability to CDCFDA and indicated knockout parasite strains with reduced permeability to CDCFDA. **D)** The percentage of CDCFDA-fluorescent vacuoles was quantified for each strain. At least 50 vacuoles per well were quantified and identified as CDCFDA-positive or negative. Data are displayed as average (±SD) values from 3 independent experiments. One-way ANOVA with Tukey’s multiple comparison test was used to determine significance (****p < .0001, n = 3). **E)** HFFs were infected with indicated parasite strains (MOI of 1) for 24 h. The parasitophorous vacuole was stained with anti-GRA5 antibody and parasites were stained with anti-SAG1 antibody and the number of parasites/vacuole was determined for ~100–200 vacuoles per experiment. Data are displayed as average (±SD) values from 3 independent experiments. A two-way ANOVA with Dunnett’s multiple comparison test was used to determine significance.

We observed that Δ*gra72* parasites formed vacuoles that have aberrant morphology (‘bubble vacuole’) (**Fig 5A–5B**). These vacuoles are grossly enlarged and a subset of them appear collapsed and contain non-dividing opaque tachyzoites similar to what we previously observed in Δ*gra17* parasites [4]. We noticed that after multiple passages the percentage of parasites forming such bubble vacuoles reduced gradually. We previously showed that Δ*gra17* bubble vacuoles have decreased permeability to small molecules [4]. To determine if the Δ*gra72* bubble vacuoles also have reduced permeability to small molecules we used the vital dye 5-(and-6)-Carboxy-2′,7′-Dichlorofluorescein Diacetate (CDCFDA), which has a molecular weight (445.2 Da) below the established size exclusion limit of the *Toxoplasma* PVM [3]. Consistent with previous results, only ~13% of Δ*gra17* vacuoles were permeable to dye at the time point examined (**Fig 5C–5D**). The vacuoles of Δ*gra72* parasites displayed a significant decrease in the dye permeability where only ~37% of vacuoles were permeable (**Fig 5D**). Complementation of the Δ*gra72* parasites with a C-terminally HA-tagged copy of *GRA72* (**S2 Fig**) rescued this phenotype. Δ*gra72* vacuoles contained significantly fewer parasites compared to vacuoles of wild-type or complemented parasites (**Fig 5E**) suggesting a slower replication of Δ*gra72* parasites. Thus, Δ*gra72* parasites phenocopy Δ*gra17* parasites.

### Alphafold-multimer predicts GRA72 to form pore like structures

**Fig 6:**
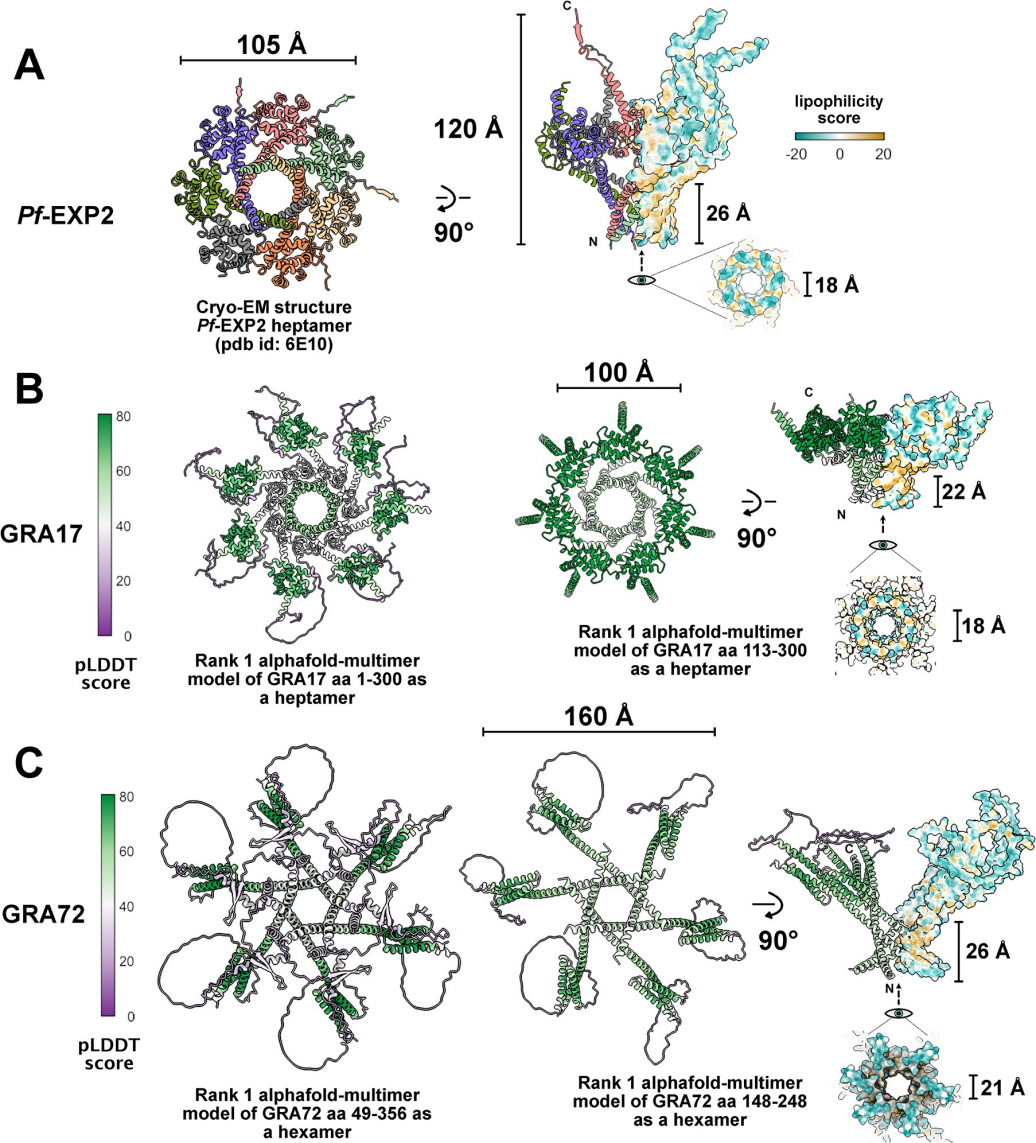
AlphaFold-multimer predicts GRA17 and GRA72 as pore like assemblies. **A**) Cartoon depiction of Pf-EXP2 structure (aa 27–235 are modelled) extracted from the full PTEX cryo-EM structure in the extended state (PDB id: 6E10). On the left, the pore forming complex is seen from the bottom and monomers are colored individually. On the right, the Pf-EXP2 complex is flipped by 90°C and depicted in a hybrid manner, with both a cartoon view with N and C-terminal ends indicated and by a lipophilicity surface representation. An under view of the pore channel is also displayed with corresponding dimensions. **B)** AlphaFold-multimer prediction of a heptameric assembly of GRA17. On the left, the full length GRA17 sequence was used for the prediction while starting on the middle and on the right, a minimized structural core (aa 113–300) was used for the prediction. Cartoon depictions are colored (from purple to green) using the predicted local distance difference test (pLDDT) produced by AlphaFold. The minimized structure on the right is also flipped by 90°C and half displayed using the surface lipophilicity representation as in panel A. N and C-terminal ends are indicated on the cartoon half while an under view of the pore channel is also displayed with corresponding dimensions. **C**) AlphaFold-multimer prediction of a hexameric assembly of GRA72 (aa 49 to 356 and aa 148 to 248) following the same representations codes as in B. All representations were produced using ChimeraX.

It was unclear why Δ*gra72* parasites formed bubble vacuoles while these vacuoles were never seen in Δ*asp5*, Δ*gra42*, Δ*gra43*, Δ*gra45*, Δ*gra57*, Δ*gra70*, and Δ*gra71* parasites, despite these parasites also exhibiting significantly reduced localization of GRA17/GRA23 to the PVM. Although we did not check the permeability of all knockout parasites, the CDCFDA permeability of Δ*gra70* parasites was like that of wild-type parasites (**S3 Fig**). Although cell permeabilization using Triton showed that GRA72 mainly localizes within the PV lumen, permeabilization with a low Saponin concentration highlighted more pronounced PVM localization (**S2 Fig**). We therefore hypothesized that GRA72, like GRA17, might also form a pore. To test the hypothesis that GRA72 could be the building block of a quaternary pore forming structure, we used AlphaFold2-multimer [32,33] through the CollabFold-mmseq2 implementation [34]. First, we iteratively ran 2/3/4/5/6/7/8-mer assemblies on GRA17 (**S4A Fig**) which bares partial sequence homology (20% sequence identity) to *Plasmodium falciparum* (*Pf*)-EXP2, the pore forming heptamer within the PTEX core complex [35]. Interestingly, as a heptameric assembly, the best AlphaFold prediction, ranked by predicted local distance difference test score (pLDDT), forms a pore like structure closely resembling the cryo-EM structure of Pf-EXP2 (**Fig 6A–6B**). This pore-like prediction occurs whether the full sequence is taken (aa1-300) or an N-terminal truncation removing a largely disordered domain (aa113-300). Next, we tested the same approach on GRA72, calculating AlphaFold-multimer predictions on a slightly truncated version of GRA72 (aa 49–356) for computational considerations. In doing so, GRA72 is also predicted to form a pore like structure with 5- or 6-fold pseudosymmetry (**Fig 6C**). Increasing the oligomers to a heptamer leads to a loosening of the funnel structure (**S4B Fig**). Similarly, as for GRA17, the pore is still consistently predicted as a hexamer when outer regions are removed (aa 148–248, **Fig 6C**).

Overall, as with *Pf*-EXP2, GRA17 and GRA72 are mostly helical proteins with a long N-terminal helix dedicated to closing the pore funnel while a C-terminal domain of alpha-helices forms a higher ring structure (**Fig 6A–6C**). Pore dimensions and features in between the predicted models and *Pf*-EXP2 are relatively similar, the observed differences can be expected from the lack of other PTEX scaffolding subunits (PTEX150 heptamer and HSP101 hexamer in *Plasmodium*) that are usually folded against the EXP2 heptamer. Of note, although all AlphaFold models assemble GRA17 in a helical ring structure, only the heptamer rank 1 had a fully formed funnel (**S4A Fig**). This non-constant fold is also reflected by the locally low pLDDT score at the funnel (**Fig 6B**), ranging from 40 to 60. Strikingly, GRA72, has an opposite thread (screw axis) when compared to *Pf-*EXP2 and GRA17 funnels (**Fig 6A–6C**).

When calculating the outer hydrophobicity surface to Pf-EXP2 (**Fig 6A**), both GRA17 and GRA72 AlphaFold-multimer predictions display a conserved 22 to 26 Å outer funnel which is highly hydrophobic and prone to interact with the phospholipid bilayer (**Fig 6A–6C**). The channel diameter of GRA17 and GRA72 also compare relatively well to the 18 Å diameter for Pf-EXP2 suggesting potential similar functions. Overall, these models suggest functional conservation between Pf-EXP2 and GRA17, while GRA72 probably forms a pore as well although strict function conservation is not as clear as we note clear differences in pseudosymmetry and overall extension of the upper ring. It is important to note that we did not observe pore-like structures when multimers of GRA57, GRA70, or GRA71 were predicted with AlphaFold (**S5 Fig**). Thus, the observation that only Δ*gra17* and Δ*gra72* parasites form ‘bubble vacuoles’ with reduced small molecule permeability seems to be associated with the ability of GRA17 and GRA72 to form pores. Future experiments are needed to confirm that GRA72 indeed forms pores in the PVM and the types of molecules that go through these pores.

### GRA72 is required for the *in vivo* growth and virulence of *Toxoplasma*

**Fig 7:**
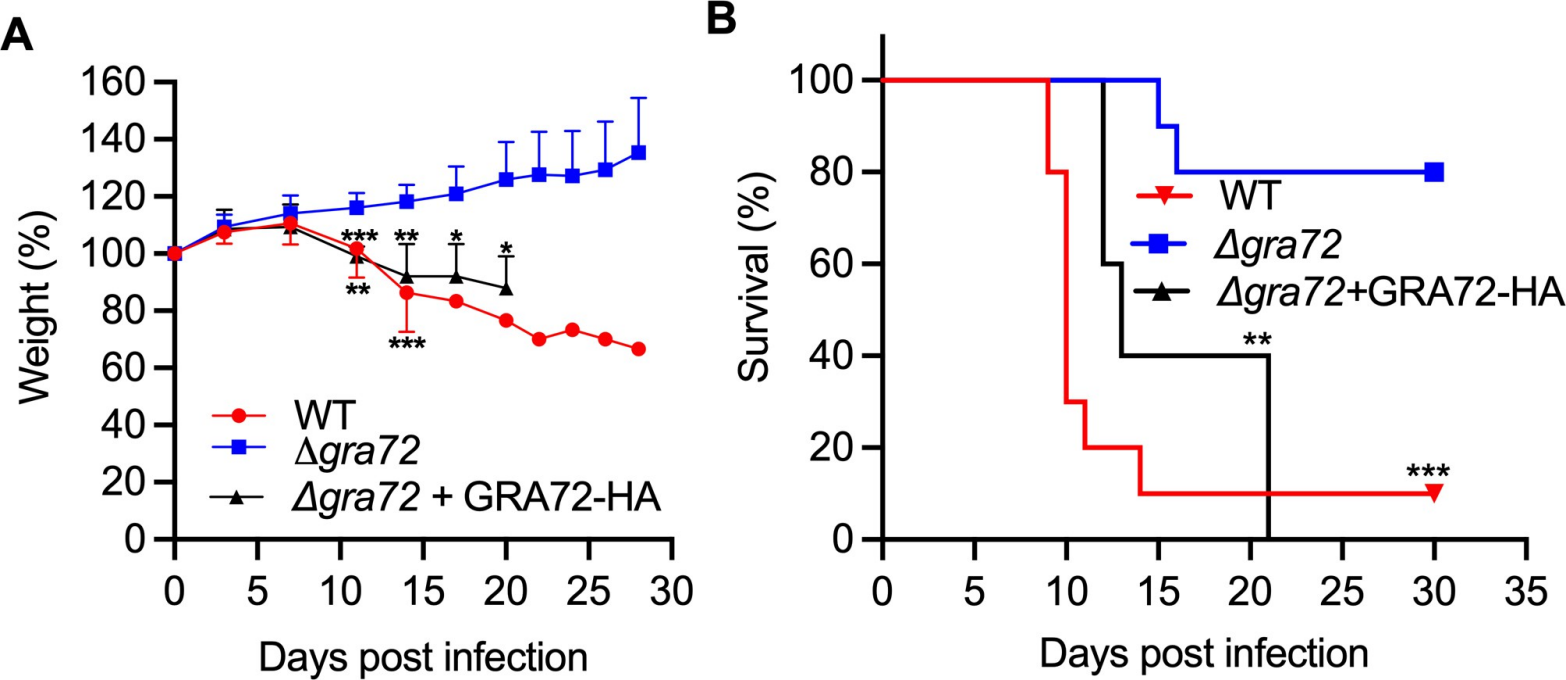
Δ*gra72* parasites have reduced *in vivo* virulence. **A.** CD1 mice were infected intraperitoneally with 5,000 tachyzoites of WT, Δ*gra72* or Δ*gra72* +GRA72-HA parasites (all in the ME49 type II strain). Mice were monitored and weighed regularly throughout the infection period and the weight is plotted as the average change in body weight for each cohort, where the weight on the day before infection was considered as 100%. The statistical significance was performed by one-way ANOVA with Tukey’s multiple comparison test from time points from day 0—day 20 (* *P*< 0.05, ** *P*<0. 01, *** *P*< 0.001). **B.** The post-infection survival of mice was monitored for 30 days. The data are displayed as Kaplan–Meier survival curves (data presented for wild-type or Δ*gra72* parasites are pooled from two independent experiments, each involving n = 5 mice, for a cumulative total of n = 10 mice, and one experiment with n = 5 for mice infected with Δ*gra72* +GRA72-HA parasites). The statistical significance was determined by the Log-rank (Mantel–Cox) test.

To examine the importance of GRA72 for *in vivo* parasite growth and virulence, we knocked out *GRA72* in the type II ME49 strain (**S6A Fig**) and intraperitoneally (i.p.) infected CD-1 outbred mice with 5,000 wild-type, Δ*gra72*, or Δ*gra72+*GRA72-HA parasites. In contrast to Δ*gra72* infected mice, mice infected with wild-type or complemented parasites showed a significant drop in body weight (**Fig 7A**) and deterioration in body condition over the duration of the experiment. Only one out of ten mice infected with wild-type parasites survived (**Fig 7B**) while eight out of ten mice infected with Δ*gra72* parasites survived throughout the duration of the experiment. All mice infected with complemented parasite strain succumbed throughout the experimental duration (**Fig 7B**). Mice infected with Δ*gra72* parasites contained few cysts (mean of 80 cysts) whereas the one surviving wild-type-infected mouse contained 750 cysts in the brain (**S7 Fig**). Δ*gra72* tissue cysts were smaller compared to cysts from the wild-type parasite strain (**S7 Fig**). However, *in vitro* cyst conversion experiments did not show a defect in Δ*gra72* cyst size or conversion rate (**S7 Fig**), suggesting that the low *in vivo* cyst burden and small *in vivo* cyst sizes are related to the reduced in vivo virulence and growth of Δ*gra72* parasites and not to inherent defects in differentiation.

## Discussion

New drug targets to treat toxoplasmosis are needed as current drugs have toxic side effects and they do not clear the chronic cyst stages [36]. The most used drug combination to treat toxoplasmosis is pyrimethamine plus sulfadiazine, which inhibit *Toxoplasma* dihydrofolate reductase (DHFR) and dihydropteroate synthase, respectively, two enzymes involved in the synthesis of tetrahydrofolates, which are essential cofactors for DNA synthesis and methionine metabolism [37]. Thus, *Toxoplasma* proteins involved in metabolism appear to be attractive targets. However, the incomplete understanding of *Toxoplasma* metabolism and its nutrient scavenging mechanisms hampers development in this area. Based on the slow growth and avirulence of Δ*gra17* parasites, GRA17 pores appear to play an important role in the import of host-derived small (<1.5 kDa) nutrients [4]. We therefore hypothesized that in the nutrient sensitized Δ*gra17* strain other parasite genes that play an important role in nutrient acquisition should become essential. In this manuscript we identified 85 genes that appear to have a larger fitness defect when knocked out in Δ*gra17* vs. wild-type parasites. We further tested 11 of these 85 hits and confirmed for four genes that they are synthetically lethal/sick with *GRA17*. Three of these genes encode for GRA proteins (GRA45, GRA70, GRA72) that affect the correct localization of GRA17/GRA23, which could explain their synthetic lethality/sickness with *GRA17*.

We previously showed that GRA45 is a chaperone-like protein, possibly shielding the hydrophobic domain of GRAs *en route* to the PVM within the secretory pathway [18]. GRA70 and GRA72, along with GRA57/GRA71, are involved in the proper localization of GRA17/GRA23, likely after their exocytosis from the dense granules into the PV lumen. In parasites with single knockouts - Δ*gra45*, Δ*gra57*, Δ*gra70*, Δ*gra71*, and Δ*gra72—*a minor fraction of GRA17 and GRA23 still seems to reach the PVM, apparently enough for their survival. However, the deletion of these genes in Δ*gra17* parasites appears lethal (or resulting in a strong fitness defect) due to a severely reduced amount of GRA23 on the PVM. We previously determined that knocking out *GRA42* (TGGT1_235870) or *GRA43* (TGGT1_237015) also leads to mislocalization of GRA17 and GRA23 [38]. Although *GRA42* and *GRA43* did not meet our stringent cut-offs for follow-up genes, their phenotype scores were significantly lower in Δ*gra17 vs*. wild-type parasites (**S1 Table**). The GRA17/GRA23 mislocalization phenotype resembles the mislocalization of PVM-localized GRAs reported in parasites with a deletion in Golgi-resident aspartyl protease (ASP)5, which cleaves the *Toxoplasma* export element (TEXEL, RRLxx) motif [28–30]. The predicted gene products from GRA57, GRA70, GRA71, and GRA72 have at least one predicted TEXEL motif. Thus, it is possible that the mislocalization of PVM-localized GRAs observed in Δ*asp5* parasites is due to a failure to cleave the TEXEL motif of one or more of these proteins. MYR4 was identified as synthetically sick with GRA17, but no role was found for MYR4 in the proper localization of GRAs. Given that we did not identify other components of the MYR export machinery, suggests the existence of an additional non-export function for MYR4.

We hypothesize that GRA42, GRA43, GRA57, GRA70 and GRA71 collectively form a transport system in the PV lumen. This system mediates GRA insertion into the PVM and prevents their insertion in the parasite plasma membrane. Alternatively, some of these GRAs might influence the lipid composition of the PVM, which could also affect the insertion of GRA proteins into the PVM.

In a recent genome-wide CRISPR screen in *Toxoplasma* grown in naive or interferon gamma (IFNγ)-treated HFFs, we found that GRA57, GRA70, GRA71, and GRA72 were important for parasite growth specifically in IFNγ-stimulated HFFs [27]. Future research will be needed to determine the exact mechanism by which these proteins determine the correct localization of PVM-localized GRA proteins and how this affects parasite resistance to IFNγ.

Interestingly, Δ*gra72* parasites formed bubble vacuoles, which were absent in Δ*gra42*, Δ*gra43*, Δ*gra45*, Δ*asp5*, Δ*gra70*, Δ*gra57*, and Δ*gra71* parasites, despite all these parasites showing significantly reduced localization of GRA17/GRA23 to the PVM. While this might be due to variations in the extent of GRA17/GRA23 mislocalization across the knockout parasites, it is more plausible that the ‘bubble vacuole’ phenotype relates to GRA72’s potential to form pores in the PVM, similar to GRA17.

We have previously shown that GRA17 is not only important for acute parasite virulence but also contributes significantly to the viability of bradyzoites within cysts [39]. Therefore, it is possible that the proteins discovered in our synthetic lethality screen to be involved in the proper localization of GRA17/GRA23 (and other GRAs) could be attractive drug candidates. Indeed, we observed that GRA72 knockout parasites were considerably less virulent in mice and formed fewer brain cysts. While this manuscript was under review, another study also identified GRA72 as important for *Toxoplasma* virulence in mice [40]. It is worth noting, however, that multiple studies [40–42] did not identify GRA57, GRA70, and GRA71 as important for *Toxoplasma in vivo* fitness. As such, it is plausible that the reduced virulence of GRA72 knockout parasites is not a result of GRA protein mislocalization in this knockout, but rather due to GRA72, like GRA17, to potentially form pores in the PVM.

GRA17 and GRA23 are orthologs of *Plasmodium falciparum* EXP2, and EXP2 can functionally complement Δ*gra17* parasites [4]. Although none of the *Toxoplasma* GRAs that are involved in the correct localization of GRA17/GRA23 are conserved in *Plasmodium* spp., it was recently shown that also in *Plasmodium* the correct localization of EXP2 to the PVM is dependent on other PVM proteins. For example, deletion of EXP1 resulted in an altered distribution of EXP2 within the PVM and in a defect in the EXP2 nutrient pore function without affecting EXP2 translocon function [43,44].

Although not further investigated in this study, it is possible that deletion of some *Toxoplasma* genes would enhance the fitness of Δ*gra17* parasites (synthetic viability). We identified 38 genes (RPKM>10) that had at least a 4-fold larger fitness defect when knocked out in wild-type compared to Δ*gra17* parasites and for which the sgRNAs were significantly enriched in Δ*gra17* vs. wild-type parasites (**S1 Table**). Three of the 38 genes are predicted to be involved in folate metabolism (TGGT1_285750 (6,7-dihydropteridine reductase or DHPR), TGGT1_305800 (6-pyruvoyl tetrahydrobiopterin synthase or PTPS), and TGGT1_266366 (a BT1 folate transporter). Possibly host-derived folate goes through GRA23 and/or GRA72 pores on the PVM and the increased expression of GRA23 and GRA72 in the Δ*gra17* parasites could make these parasites less dependent on folate synthesis by the parasite.

A limitation of our data is that we only performed the genome-wide CRISPR screen in Δ*gra17* parasites once. The main reason for this was that Δ*gra17* parasites already have a significant decrease in viability compared to wild-type parasites. This is likely because we previously showed, using live imaging, that many of the Δ*gra17* bubble vacuoles collapse, which caused parasite death inside the vacuole [4]. The decrease in viability of Δ*gra17* parasites made it challenging to maintain the complexity of the mutant pool and resulted in random loss of mutants. As a consequence, our list of screen hits might contain false positives. In addition, the stringent criteria we established for gene inclusion in our hit list could potentially result in the exclusion of some relevant genes, leading to false negatives. Furthermore, our approach for confirming screen hits was designed to rapidly test multiple candidates from the CRISPR screen. However, this setup is likely not as sensitive as making double knockouts and comparing their growth phenotype to single knockouts. For example, even though the transfection of Cas9 and 2 sgRNAs targeting hits from the screen (specifically *GRA70* and *GRA72*) in Δ*gra17* parasites resulted in plaque formation, we were unable to generate double knockout parasites. Analysis of the parasites that grew out of these plaques revealed that the double-stranded DNA breaks mediated by CRISPR/Cas9 were repaired in a manner that did not lead to the creation of a defective protein. Thus, future studies should consider repeating the synthetic lethality screen with a more focused sgRNA library (for instance, targeting only the top hits from this screen), or should test more hits from our screen.

Overall, using a genome-wide CRISPR screen we identified the *Toxoplasma* genes that specifically impact parasite viability when deleted in Δ*gra17* parasites. This led to identification of several novel *Toxoplasma* GRAs that are involved in correct trafficking of GRA17/GRA23 to the PVM and the identification of GRA72 that is predicted to form pores in the PVM. Because many *Toxoplasma* genes have unknown functions, performing similar synthetic lethality screens as reported here might provide insights into the biological functions of these genes.

## Materials and methods

### Host cells and parasites

Human foreskin fibroblasts (HFFs) were cultured under standard conditions using Dulbecco Modified Eagle Medium (DMEM) with 10% fetal bovine serum (FBS), 2 mM L-Glutamine, 100 U/mL Penicillin/Streptomycin, and 10 μg/mL Gentamicin. The *Toxoplasma* type I (RH) strain engineered to constitutively express Cas9 (RH Cas9 Δ*hxgprt*) [24] was used to make RHCas9*Δgra17*. Individual *GRA17* (TGGT1_222170) knockout parasites were generated using the CRISPR-Cas9 technique with single guide (sg)RNA sequences targeting *GRA17* that were cloned into the pU6-Universal vector (Addgene #52694)[45]. Plasmids containing sgRNAs were co-transfected with XhoI (New England Biolabs)-linearized pTKOatt, which contains the *HXGPRT* selection cassette [46], into parasites at a ratio 5:1 (sgRNAs: linearized pTKOatt plasmid). After 24 h, the populations were selected with mycophenolic acid (25 μg/ml) and xanthine (25 μg/ml) and cloned by limiting dilution. Gene disruption was assessed by PCR using the primers listed in **S3 Table**. The above strains were used to perform the whole genome synthetic lethality screen and to generate single knockouts of the selected genes.

### *Toxoplasma gondii* CRISPR-Cas9 mediated genome-wide loss-of-function screens

A library of sgRNAs containing ten different sgRNAs against each of 8,156 *Toxoplasma* genes was used to perform a genome-wide loss-of-function screen according to a previously published protocol [47]. Before performing the genome-wide loss-of-function screen, the efficiency of the disruption of the *SAG1* gene was confirmed to be at least 97% by transfecting RH-Cas9 with pU6-SAG1-DHFR (Addgene, Cat. No. 80322). The sgRNA library plasmids were linearized using AseI and 500 μg plasmid was divided into 5 electroporation cuvettes containing 1x10^8^ parasites each. HFFs were infected with parasites (MOI = 0.5) following plasmid electroporation (Gene Pulser, Bio-Rad) at 25 mFD, 1250 V and ∞ Ω and the parasites were grown for 24 h In medium containing DMEM with 40 μM Chloramphenicol (CAT), 1% FBS, 1% penicillin/streptomycin and 2 mM L-Glutamine. 24 h after infection, the medium was removed and replaced with DMEM containing 10% FBS, 1 μM Pyrimethamine, 40 μM CAT Chloramphenicol, 10 μg/mL gentamicin, 100 U/mL Penicillin/Streptomycin, 1 mM Sodium Pyruvate, 1x Non-Essential Amino Acids, 10 mM HEPES, and 2 mM L-Glutamine, and 10 μg/mL DNase I. Parasites were harvested from host cells by syringe lysis once large vacuoles were formed and 1x10^7^ parasites were pelleted and collected for genomic DNA extraction while another 2x10^7^ parasites were used to infect a new monolayer of HFFs. After each passage, parasites were harvested and the genomic DNA extracted using the DNeasy Blood and Tissue kit (QIAGEN). To determine relative sgRNA abundance, sgRNAs were amplified with primers P5 and P7 and sequenced with a NEXT Seq (Illumina) with single-end reads using primers (P150 and P151) (**S3 Table**).

### Bioinformatic analysis of the loss-of-function screens

Custom scripts were used to analyse the CRISPR screen [24,47] whereas statistical analysis of the CRISPR screen data was performed by Excel and R (www.R-project.org). The sgRNA library was used as a reference to match the Illumina sequencing reads. The abundance of each sgRNA sequence was calculated and normalized to the total number of matched reads. sgRNAs with zero reads were given a pseudo-count matching to 90% of the lowest value in that sample (raw sgRNA count data are shown in **S4 Table**). To determine the “phenotype” or “fitness” score, the mean log2 fold change for the top five scoring guides were calculated (**S1 Table**), which minimises the effect of stochastic losses and reduces the variation between biological replicates. To identify the genes that underwent negative or positive selection, the raw read numbers for all ten sgRNAs between two samples were compared and the negative and positive selection P-value for each gene was calculated using the MAGeCK algorithm [48] (**S1 Table**).

### Confirmation of genes potentially synthetic lethal with *GRA17*

We selected 11 genes from the 85 hits from the genome-wide screen based on the following criteria: RPKM >20, ToxoDB phenotype score > 0. From that list of 43 genes, we mostly picked genes encoding for dense granule proteins but also added some random non-secretory genes. To confirm these 11 hits identified from the screen, RH-Cas9 (wild-type) and RH-Cas9Δ*gra17* (Δ*gra17*) parasites were transfected with 2 plasmids, each containing a different sgRNA targeting the 11 genes under investigation. Immediately after transfection plaque assays were performed by adding 5,000 parasites into 3 wells of a 6-well plate with HFFs in the presence of 1 μM pyrimethamine (Sigma–Aldrich, Cat#46706) (the plasmid contains a pyrimethamine resistance cassette). Plaque numbers were determined 8 days p.i. As a negative control, we used sgRNAs targeting *SAG1*, of which the knockout has no phenotype in either background, while as positive controls we used sgRNAs targeting *GRA23* (TGGT1_297880), which is synthetically lethal with *GRA17* but of which the knockout has no growth effect in wild-type [4]. sgRNAs targeting *CDPK1* (TGGT1_301440, phenotype score = -3.3), which was previously shown to be essential in RH [23], and TGGT1_223440 (phenotype score = -3.6 [24]) were used as the control for genes important for fitness in both wild-type and Δ*gra17* parasites.

### Plaque assay

HFFs were seeded and grown in 24 well plates and the confluent monolayers were infected with different parasite strains. 100 parasites were used to infect each well. Five days post infection, plaque areas were imaged and captured using the 4x objective of a Nikon TE2000 inverted microscope equipped with Hamamatsu ORCA-ER digital camera. From each well the area of at least 25 plaques were measured and the mean plaque area of at least two wells was determined (two technical replicates for each strain). Plaque areas were measured using ImageJ software and the data were analysed by GraphPad prism.

### Generation of single and double knockouts, complemented, and endogenously tagged parasite strains

To make the TGME49_272460 (*GRA72*) knockout strain in the type II background, an ME49 strain expressing Red Fluorescent Protein (RFP) was used. The pU6-Universal plasmid containing a sgRNA against the gene of interest together with NotI (New England Biolabs)-linearized pTKOatt, which contains the *HXGPRT* selection cassette and Green Fluorescent Protein (GFP), were co-transfected into ME49 RFP+ Δ*hxgprt* parasite at a ratio of 5:1 (sgRNAs: linearized plasmid). The transfected parasite strains were selected with 25 μg/ml mycophenolic acid (MPA) (Millipore 89287) and 25 μg/ml xanthine (Xan) (Millipore X3627). Individual knockout clones were isolated by limiting dilution after three rounds of drug selection with MPA-Xan and confirmed by PCR (**S6 Fig**) using primers indicated in **S3 Table**.

To generate *GRA72* knockout parasites in the type I background, RH-Cas9 parasites were transfected with the pU6-DHFR vector (Addgene, #80329) containing a sgRNA against *GRA72* and a dihydrofolate reductase (DHFR) resistance cassette. The parasites were selected with 1 μM pyrimethamine 24 h post-transfection and after three rounds of selection, single clones were isolated by limiting dilution. The positive knockout parasite clones were confirmed by PCR. *GRA57*, *GRA70*, *GRA71* knockout parasites and the complementation of *GRA70* knockout parasites were previously described [27].

To complement the ME49 Δ*gra72* parasites, the 5’ upstream (1000 bp), coding sequence, HA epitope tag before stop codon and 3’ downstream fragments (500 bp) were cloned into the pTwist CMV vector (Twist Biosciences) ([49–51]. Δ*gra72* parasites were transfected with the pU6-Universal plasmid containing sgRNAs that target the *UPRT* locus and the complementation construct at a ratio of 5:1 (sgRNAs: complementation plasmid). After the parasites lysed out, they were selected with 10 μM 5-fluoro-2-deoxyuridine (FUDR) (Sigma) for three passages. Single clones were isolated by limited dilution and confirmed by Western blotting and immunofluorescence assay (**S2 Fig**).

To generate *GRA45* single knockout parasite strains, plasmids containing sgRNAs were co-transfected with linearized pTKO, which contains the *HXGPRT* selection cassette, into RH-Cas9 Δ*hxgprt* parasites. The parasites were selected with MPA-Xan 24 h post transfection. Δ*gra17*Δ*gra45* double knockout parasites were generated in the RH-Cas9 Δ*gra17* HXGPRT+ background by co-transfecting plasmids containing sgRNAs targeting the *GRA45* locus along with purified amplicons containing a pyrimethamine-resistant (DHFR*) cassette. Individual knockout clones were grown in medium supplemented with 3 μM pyrimethamine and single clones isolated by limiting dilution.

The *MYR4* single knockout was generated by co-transfecting plasmids containing sgRNAs targeting the *MYR4* locus with an amplicon harbouring the DHFR* cassette into RHCas9 Δ*hxgprt* parasites and individual knockout clones were isolated as mentioned above. Δ*gra17*Δ*myr4* double knockout parasites were generated in the RHCas9 Δ*gra17* HXGPRT+ background using a DHFR* cassette following similar methods as described for generation of the Δ*myr4* single knockout.

Endogenous tagging was performed in the RHΔ*ku80*Δ*hxgprt* [52] parasite background. To introduce an HA epitope tag at the C-terminus of the GOI, a donor template was made by PCR amplification of 3xHA-3’UTR followed by the *DHFR* cassette from the pLIC plasmid [52]. The donor template contained a 5′ region of 40 base pair homology chosen from the sequence immediately upstream of the stop codon of the GOI in-frame with 3xHA tag followed by a stop codon. The 3′ region of 40 base pair homology was chosen from the GOI downstream of the CRISPR cut side. The pU6-Universal plasmid containing a sgRNA against the gene of interest and donor template were electroporated into *Toxoplasma* at a ratio of 5:1. Transfected parasite populations were cloned by limiting dilution to isolate single clones. The presence of GRA72-HA was confirmed by immunofluorescence assays and Western blotting (**S2 Fig**). A rat anti-HA IgG antibody diluted 1:500 in 5% (w/v) skim milk in PBS-T was used as a primary antibody and the secondary antibody (anti-rat IgG HRP) diluted 1:10,000 in 1% (w/v) skim milk in PBS-T was used for staining.

### Parasites per vacuole counting

HFFs were grown in 24 well plates containing coverslips and infected with parasites (MOI of 1) harvested by syringe lysing. The plates were centrifuged for 2 minutes at 162 x g and incubated at 37°C in a CO_2_ incubator for 24 h. The coverslips were fixed with 3% formaldehyde for 20 minutes and blocked for 1 h using a blocking buffer consisting of 3% (w/v) BSA, 5% (v/v) goat serum and 0.1% Triton X-100 in PBS. Mouse anti-GRA5 (1:500) antibody was used to detect the parasitophorous vacuole and rabbit anti-SAG1 antibody (1:4000) was used to stain the parasites inside the vacuole. Secondary antibodies anti-mouse Alexa Fluor (594) and anti-rabbit Alexa Fluor (488) were used at a dilution of 1:3000. DAPI was used to stain nucleic acids. For 100–200 vacuoles per experiment the number of parasites per vacuole was determined.

### Live cell imaging of PVM permeability

HFFs were grown on glass-bottom dark 24-well plates (Greiner Bio-One) and the confluent monolayers were infected with different parasite strains at an MOI of 1 in DMEM containing 1% FBS. At 24 h post-infection, the cells were washed with PBS after which Gibco DMEM/F-12 (Invitrogen) medium without phenol red and supplemented with 10 μM 5(6)-Carboxy-2’,7’-dichlorofluorescein diacetate (CDCFDA) was added to the cells. After 10 minutes of incubation at 37°C, the medium was removed and the cells were washed three times with PBS. Gibco DMEM/F-12 growth medium was added to the cells and the cells were imaged immediately.

### Host GFP ingestion assay

A previously published ingestion assay was followed [9]. One day before transfection Chinese hamster ovary (CHO) cells were seeded in 6-well plates to achieve a 60–75% confluency. Confluent CHO cells were transfected with 2 μg GFP-expressing plasmid using a 3:1 ratio of transfection reagent (XTREME GENE 9) to μg plasmid DNA. A 24 h post-transfection, the cells were infected by parasites treated or not by the Cathepsin L (CPL) inhibitor, morpholine urea-leucyl-homophenyl-vinyl sulfone phenyl (LHVS) (kind gift from Dr. Matthew Bogyo, Stanford University) at a final concentration of 1 μM. One day after infection, the parasites were harvested by syringe lysing and filter purified on ice. Harvested parasites were pelleted by centrifuging at 4°C for 10 minutes at 1,000 x g. The supernatant was aspirated and resuspended in Saponin/Pronase solution followed by incubation for 1 h at 12°C in the immersion circulator. The solution was washed by ice cold PBS again and centrifuged at 4°C for 10 minutes at 1,000 x g. The supernatant was taken and the parasite suspension was deposited onto Cell-Tak coated glass slide. The parasites were fixed with 4% Formaldehyde and permeabilized with 0.1% Triton X-100, stained with mouse anti-CPL antibody. Following antibody staining, the parasites were imaged by Nikon TE2000 inverted microscope equipped with Hamamatsu ORCA-ER digital camera.

### PVM GRA localization

To investigate the localization of GRA17 and GRA23, different parasite strains transiently expressing GRA17-HA, GRA17-V5, or GRA23-HA were used to infect confluent HFFs seeded in 24 well plates containing coverslips at an MOI of 0.5. At 24 h post-infection, the cells were fixed with 3% Formaldehyde in PBS for 20 minutes followed by incubation with blocking buffer (3% (w/v) BSA, 5% (v/v) goat serum and 0.1% Triton X-100 in PBS) at room temperature for 1 h. Primary antibodies rat anti-HA, rabbit anti-SAG1, and mouse anti-V5 (Thermo Scientific, MA5-15253) diluted at 1:500, 1:4000, and 1:1000 in blocking buffer, respectively, were used to stain the cells. Following overnight incubation with primary antibodies at 4°C, the coverslips were stained with goat anti-rat Alexa Fluor 594 (Thermo Scientific, A-11007), goat anti-mouse Alexa Fluor 594 (Thermo Scientific, #A11032) and Goat anti-rabbit Alexa Fluor 488 (Thermo Scientific, #A11008) diluted in blocking buffer at 1:3000. DAPI diluted at 1:2000 was used to stain DNA. The coverslips were mounted using Mowiol mounting medium and pictures were taken under an epifluorescence inverted microscope Nikon (eclipse Ti-S; Nikon) connected to NIS-Elements software (Nikon) using a digital camera (CoolSNAP EZ; Roper Scientific). To compare the localization of GRA23 and GRA17, at least 50 vacuoles containing four or more parasites were quantified and GRA17/GRA23 categorized as PVM localized, partially PVM localized or PV lumen localized. To determine localization of GRA5 and GRA7, HFFs were seeded in 24 well plates and infected with parasite strains at MOI of 0.5 for 24 h. The cells were fixed with 3% formaldehyde for 20 minutes, blocked with PBS containing 3% (w/v) BSA, 5% (v/v) goat serum and 0.1% Saponin. The coverslips were incubated and stained with mouse anti-GRA5 (red), rabbit anti-SAG1 (green), rabbit anti-GRA7(red), mouse anti-SAG1 (green) antibodies and DAPI was used to stain nucleic acids. Secondary antibodies goat anti-mouse Alexa Fluor 594 (Thermo Scientific, #A11032), Goat anti-rabbit Alexa Fluor 488 (Thermo Scientific, #A11008), Goat anti-rabbit Alexa Fluor 594 (Thermo Scientific, #A11037) and Goat anti-mouse Alex Fluor 488 (Thermo Scientific, #A11029) were diluted in a blocking buffer at 1:3000 to stain coverslips together with DAPI. The percentage of vacuoles with PV lumen or PVM GRA5 and GRA7 staining were quantified.

### *In vivo* infection and brain cyst counting

*Toxoplasma* tachyzoites from wild-type (ME49 RFP+ Δ*hxgprt*), ME49 Δ*gra72*, and ME49 Δ*gra72 +GRA72-HA* parasites were harvested from HFFs by syringe lysing through a 27-gauge needle. CD-1 mice at the age of six weeks were injected intraperitoneally with 5,000 parasites. Parasite viability was determined by plaque assay immediately after infection of the mice. The mice were monitored daily for 30 days and weighed every two days. 30 days post infection the mice were sacrificed, and brains were harvested for cyst isolation. Mouse brains were homogenized in PBS and 1/10^th^ of the homogenate was fixed with ice cold methanol. Tissue cysts were stained with DBA-FITC (FL-1031-5 Vector Laboratories) at 1:500 dilution and imaged with a digital camera. Cyst areas were measured by ImageJ software.

### *In vitro* stage differentiation

Differentiation to encysted bradyzoites was performed as previously described [53]. Cyst sizes of Dolichos biflorus Agglutinin (DBA)-positive cysts were measured for at least 30 cysts in three independent experiments. Conversion was calculated by determining the fraction of PVs positive for SAG1 (tachyzoite marker), SAG2Y (bradyzoite marker), or both.

### AlphaFold2-Multimer structure predictions

AlphaFold2-multimer [32] predictions were run through the ColabFold/Mmseqs2 [34] workflow (v 1.3.0) on an Nvidia A5000 graphics card, which enables multimer calculations up to 2300–2500 total amino acids (depending on input sequences and multimerization factor). Key input parameters were as follows: use_templates: “false”, use_amber: “false”, msa_mode: “Mmseqs2 (UniRef+Environmental)”, model_type: “AlphaFold2-multimer-v2”, num_models: “5”, num_recycles: “3”, num_ensemble: “1”, keep_existing_results: “true”, rank_by: “multimer”, max_msa: “null”, pair_mode: “unpaired+paired”, stop_at_score: “100”, stop_at_score_below: “0”. All models were depicted using UCSF ChimeraX [54].

### Statistical analyses

Statistical analyses were performed using GraphPad prism. ANOVA was used when three or more groups were compared. A one-way ANOVA with Tukey’ multiple comparisons test was used to compare the data that has three or more groups and only one independent variable or factor. A two-way ANOVA with Dunnett’s multiple comparison test was used to compare three or more groups with two independent variables. To compare the statistical significance and difference between two groups, a t-test was used. For any statistical tests, *p < 0*.*05* was considered as significant. The Log-rank (Mantel–Cox) test was used to analyze the mouse survival experiment. The data are presented as mean *±* standard deviation. All the data presented are from three or more independent experiments and the n values are mentioned in each figure legend.

## Supporting information

S1 Table Phenotypic impact of *Toxoplasma* gene knockout in wild-type vs. Δ*gra17* parasites. This table contains the phenotype scores for each *Toxoplasma* gene when knocked out in either wild-type (passage 1, 3, 4 and 8) or Δgra17 parasites (passage 1, 3, and 4). It further provides P-values that compare presence of sgRNAs at different passages in RH vs. Δgra17 parasites. Moreover, this table contains the FPKM (Fragments Per Kilobase of transcript per Million mapped reads) values derived from RNAseq data for the following strains: RHΔ*ku80*Δ*hxgprt*, RHΔ*ku80*Δ*gra17*, RHΔ*ku80*Δ*gra17*+GRA17_[low expression]_, and RH GRA17_overexpression_.
(XLSX)

S2 Table Pathway enrichment analysis of potentially synthetically lethal/sick *Toxoplasma* genes with GRA17. This table contains the results of the pathway enrichment analysis of 85 identified *Toxoplasma* genes that meet the criteria for potential synthetic lethality or sickness in combination with GRA17. Employing resources from ToxoDB, the table evaluates enrichment within both Metabolic pathways and Gene Ontologies. It highlights significantly overrepresented Gene Ontologies or Metabolic pathways. These enrichments provide insight into the broader biological functions and processes these genes may be involved in, and suggest potential interplay between these genes and GRA17.
(XLSX)

S3 Table Catalogue of primers and antibodies used in this study. This table lists the primers and antibodies used in our study. It includes the specific primers used for Illumina sequencing of the sgRNAs, which were amplified from the Toxoplasma parasite’s DNA. It also lists the oligonucleotides that were used to generate sgRNA constructs used to knockout specific *Toxoplasma* genes. In addition, the table lists other primers that were used in this study for various experimental procedures. Furthermore, it details the antibodies that were used.
(XLSX)

S4 Table sgRNA read counts in wild-type and Δ*gra17* parasites at different passages. This table contains the read counts for each unique sgRNA detected in wild-type and Δ*gra17* parasites at different passages. These data were subsequently used to calculate a phenotype score for each *Toxoplasma* gene in wild-type and Δ*gra17* parasites.
(XLSX)

S1 Fig GRA72 does not influence the export of GRA16 and GRA24. HFFs were infected with either WT, Δ*gra72* knockout, or complemented parasites transiently expressing GRA16-Ty or GRA24-Ty. Twenty-four h p.i., the cells were fixed with 3% formaldehyde for 20 minutes and stained with mouse anti-Ty (red) antibody. **A&B)** Representative images of GRA16 and GRA24 exported to the host nucleus, respectively. **C&D)** Quantification of the nuclear intensity of GRA16 and GRA24 from A and B, respectively. Statistical analysis was performed using a one way-ANOVA with Tukey’s multiple comparison test. Shown in colored dots are averages from 5 independent experiments, black dots represent data from individual nuclei. The images are representative of five independent experiments and the scale bars represent 10 μm. ns = not significant.
(TIF)

S2 Fig Confirmation of endotagged and complemented strains. **A)** A Western blot showing the complementation of the Δ*gra72* knockout in the type 1 (RH) and type 2 (ME49) background or C-terminally HA endotagged GRA72. The GRA72 protein has a molecular weight of 51.6 kDa and was detected with an antibody against the HA-tag. **B)** An IFA showing the localization of GRA72 from C-terminally HA tagged or from complemented Δ*gra72* parasites (in red) using different permeabilizations. Scale bar = 7μM.
(TIF)

S3 Fig Δ*gra70* parasites have normal PVM permeability to small molecules. **A)** HFFs were infected with indicated parasite strains for 24 h and subsequently pulsed with CDCFDA for 10 minutes after which the dye was washed away and vacuoles were imaged. Shown are representative images from the wild-type and Δ*gra70* parasite strains showing normal permeability to CDCFDA and the Δ*gra17* parasite strains with reduced permeability to CDCFDA. **B)** The percentage of CDCFDA-fluorescent vacuoles was quantified for each strain. At least 50 vacuoles per well were quantified and identified as CDCFDA-positive or negative. Data are displayed as average (±SD) values from 3 independent experiments. One-way ANOVA with Tukey’s multiple comparison test was used to determine significance (****p < .0001, n = 3).
(TIF)

S4 Fig AlphaFold-multimer incremental homo-oligomeric predictions of GRA17 and GRA72. **A**) 3/4/5/6/7/8 mer predictions of GRA17 (aa 1 to 300). **B**) 3/4/5/6/7 mer predictions for GRA72 (aa 49 to 356). The rank 1 model (out of 5) by pLDDT score is shown in all cases. Complexes are displayed in a cartoon fashion using ChimeraX and colored by chain.
(TIF)

S5 Fig AlphaFold-multimer incremental homo-oligomeric predictions of helical domains of GRA57, GRA70 and GRA71. **A**) 3/4/5/6/7 mer predictions of GRA57 (aa306 to 557). **B**) 3/4/5/6 mer predictions of GRA70 (aa131 to 374). **C**) 3/4/5 mer predictions of GRA71 (aa290 to 709). The rank 1 model (out of 5) by pLDDT score is shown in all cases. Complexes are displayed in a cartoon fashion using ChimeraX and colored by chain.
(TIF)

S6 Fig Generation of knockout parasite strains. Shown in **A)** is the schematic diagram of the strategy used to delete *GRA72* in the type 2 strain and indicated by a red box is the CRISPR/Cas9-targeting site. Linearized pTKO plasmid carrying a GFP and an HXGPRT selection cassette was used as a repair template and the selection was performed with mycophenolic acid and xanthine. **B)** Disruption of the gene of interest (GOI) was confirmed with primers P1 and P2 amplifying a region in the GOI while MYR4 was used as a PCR control. Insertion of a repair template was confirmed with primers P1+P3. **C)** Shown is the schematic diagram of the strategy used to delete a GOI in RH-Cas9 Δ*hxgprt* or RH-Cas9 Δ*gra17* parasites using either a DHFR or HXGPRT resistance cassette. **D)** Disruption of the GOI was confirmed with primers P1 and P2. Insertion of a repair template was confirmed by primers P1+P3/P4 or P2+P3.
(TIF)

S7 Fig Δ*gra72* parasites form fewer cysts in mice but have no *in vitro* conversion defect. Surviving CD-1 mice from **Fig 7** were sacrificed and the number and size of cysts was quantified. **A)** Representative images of wild-type or Δ*gra72* cysts. Scale bars indicate 10 μm. **B)** Cyst area was measured from wild-type (n = 10 cysts) and Δ*gra72* (n = 4 cysts) cysts. Statistical significance was determined by unpaired t-test and error bars indicate SD. **C)** Number of cyst/brain 30 days post infection, brains were isolated from wild type (n = 1) or Δ*gra72* (n = 8) infected mice and cyst numbers were quantified. **D**) Indicated parasites (in the ME49 type II strain) were converted to *in vitro* cysts and after 14 days cells were fixed and the cyst wall was stained with Dolichos biflorus agglutinin. Cyst size was quantified from at least 30 cysts per experiment. Shown are the average cyst sizes for 3 independent experiments. One-way ANOVA with Tukey’s multiple comparison test was used to determine significance (ns = not significant). **E**) Parasites were converted as in D but after fixation parasites were stained with antibodies against the bradyzoite SAG2Y and tachyzoite SAG1 surface markers. The percentage of vacuoles with at least 4 or more parasites that stained for the indicated surface markers is indicated. Shown are averages and SD from 3 independent experiments.
(TIF)
