# Supplementary figures and images for: Genome-wide CRISPR screen identifies genes synthetically lethal with GRA17, a nutrient channel encoding gene in Toxoplasma
Source: PLoS Pathog. 2023 Jul 27;19(7):e1011543. doi: 10.1371/journal.ppat.1011543 (PMC10409377; doi:10.1371/journal.ppat.1011543)

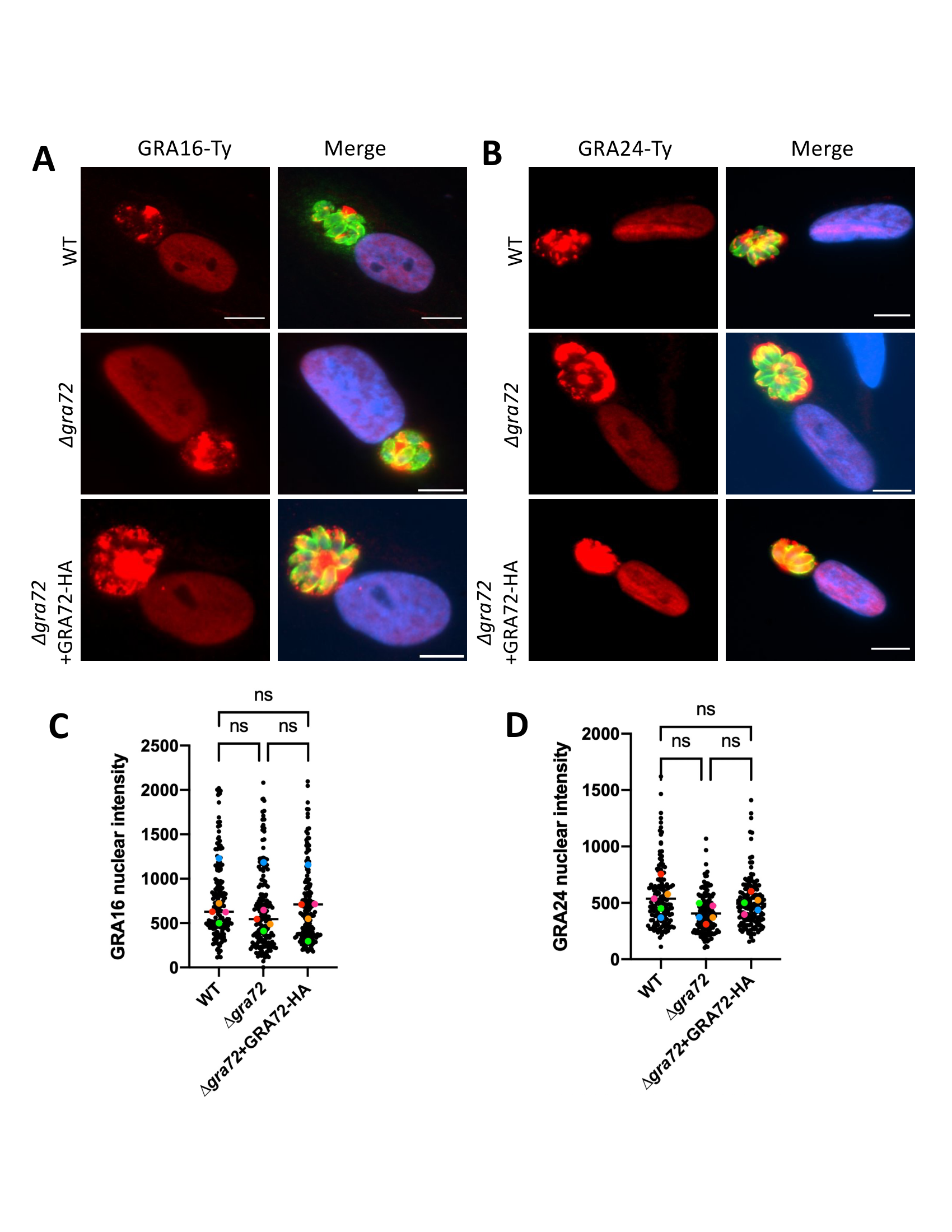

Supplement: S1 Fig — HFFs were infected with either WT, Δgra72 knockout, or complemented parasites transiently expressing GRA16-Ty or GRA24-Ty. Twenty-four h p.i., the cells were fixed with 3% formaldehyde for 20 minutes and stained with mouse anti-Ty (red) antibody. A&B) Representative images of GRA16 and GRA24 exported to the host nucleus, respectively. C&D) Quantification of the nuclear intensity of GRA16 and GRA24 from A and B, respectively. Statistical analysis was performed using a one way-ANOVA with Tukey’s multiple comparison test. Shown in colored dots are averages from 5 independent experiments, black dots represent data from individual nuclei. The images are representative of five independent experiments and the scale bars represent 10 μm. ns = not significant. (TIF) [file ppat.1011543.s005.tif]

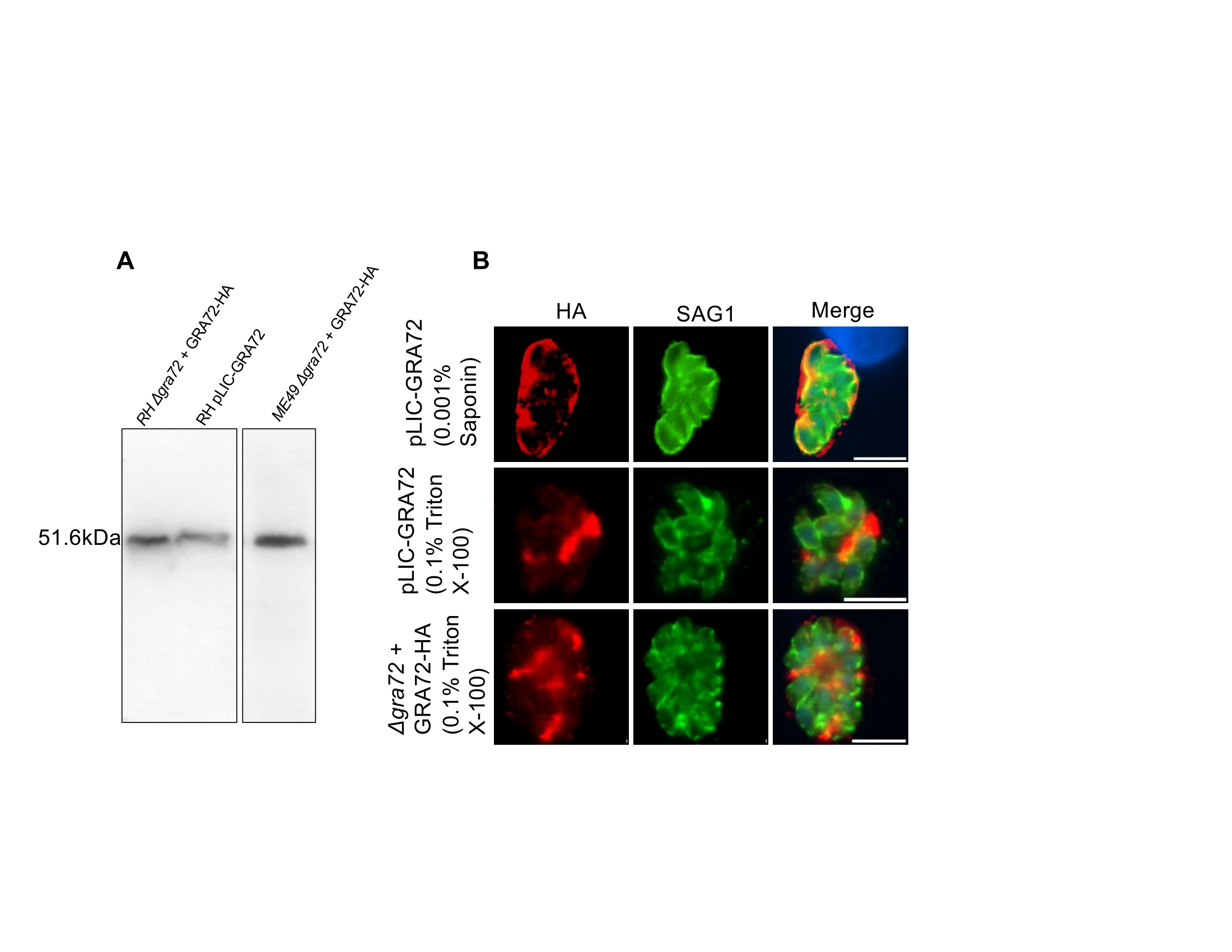

Supplement: S2 Fig — A) A Western blot showing the complementation of the Δgra72 knockout in the type 1 (RH) and type 2 (ME49) background or C-terminally HA endotagged GRA72. The GRA72 protein has a molecular weight of 51.6 kDa and was detected with an antibody against the HA-tag. B) An IFA showing the localization of GRA72 from C-terminally HA tagged or from complemented Δgra72 parasites (in red) using different permeabilizations. Scale bar = 7μM. (TIF) [file ppat.1011543.s006.tif]

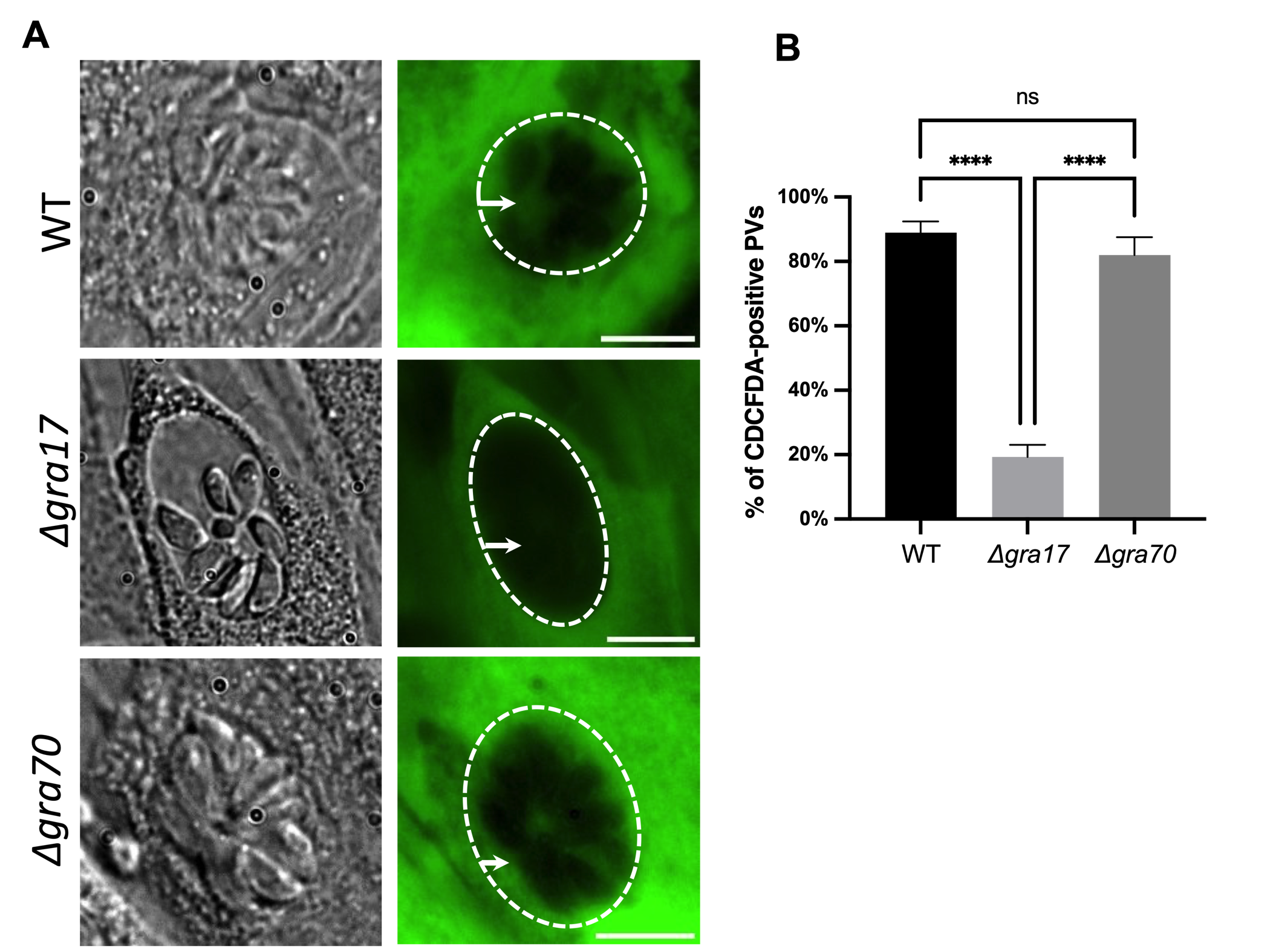

Supplement: S3 Fig — A) HFFs were infected with indicated parasite strains for 24 h and subsequently pulsed with CDCFDA for 10 minutes after which the dye was washed away and vacuoles were imaged. Shown are representative images from the wild-type and Δgra70 parasite strains showing normal permeability to CDCFDA and the Δgra17 parasite strains with reduced permeability to CDCFDA. B) The percentage of CDCFDA-fluorescent vacuoles was quantified for each strain. At least 50 vacuoles per well were quantified and identified as CDCFDA-positive or negative. Data are displayed as average (±SD) values from 3 independent experiments. One-way ANOVA with Tukey’s multiple comparison test was used to determine significance (****p < .0001, n = 3). (TIF) [file ppat.1011543.s007.tif]

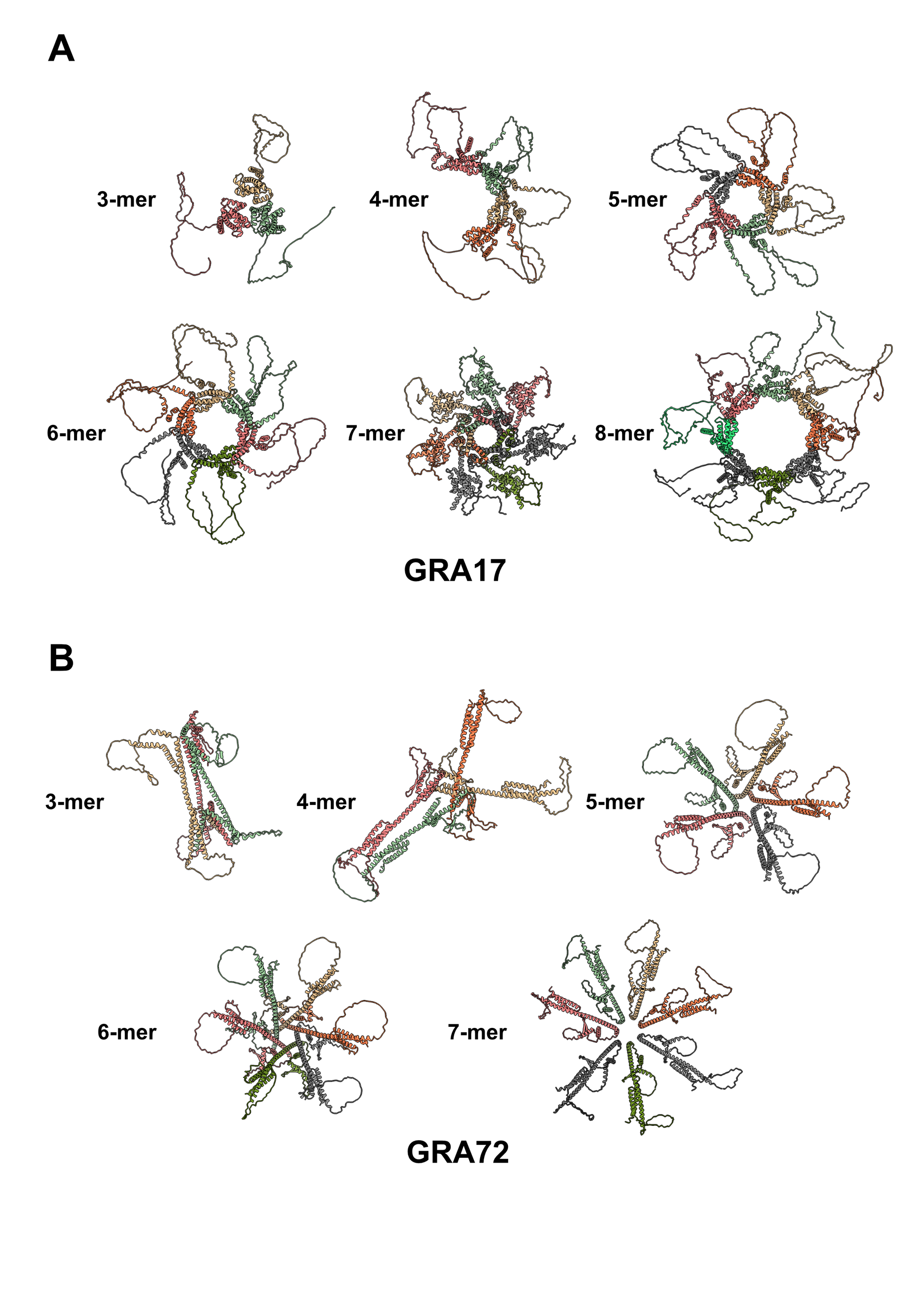

Supplement: S4 Fig — A) 3/4/5/6/7/8 mer predictions of GRA17 (aa 1 to 300). B) 3/4/5/6/7 mer predictions for GRA72 (aa 49 to 356). The rank 1 model (out of 5) by pLDDT score is shown in all cases. Complexes are displayed in a cartoon fashion using ChimeraX and colored by chain. (TIF) [file ppat.1011543.s008.tif]

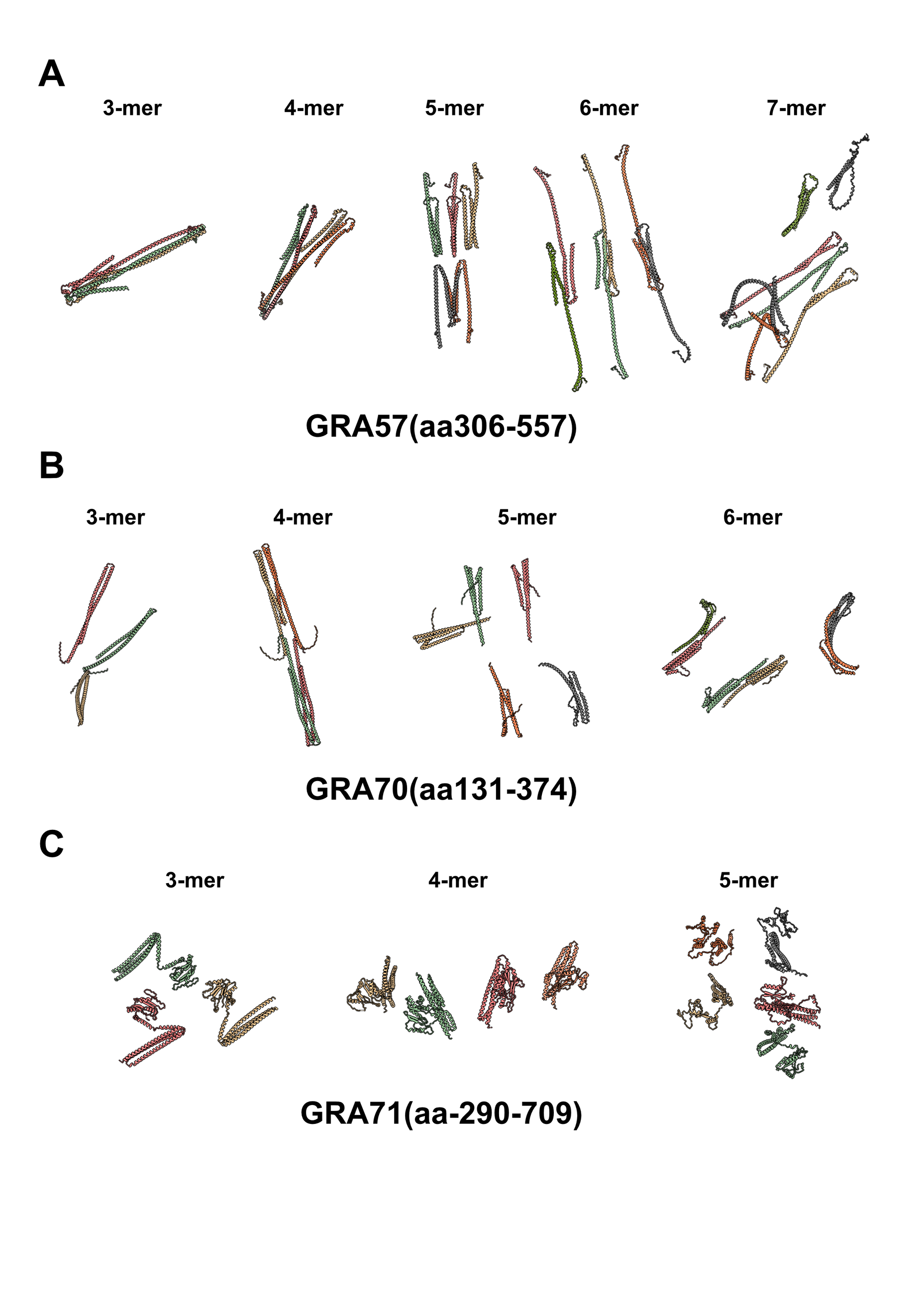

Supplement: S5 Fig — A) 3/4/5/6/7 mer predictions of GRA57 (aa306 to 557). B) 3/4/5/6 mer predictions of GRA70 (aa131 to 374). C) 3/4/5 mer predictions of GRA71 (aa290 to 709). The rank 1 model (out of 5) by pLDDT score is shown in all cases. Complexes are displayed in a cartoon fashion using ChimeraX and colored by chain. (TIF) [file ppat.1011543.s009.tif]

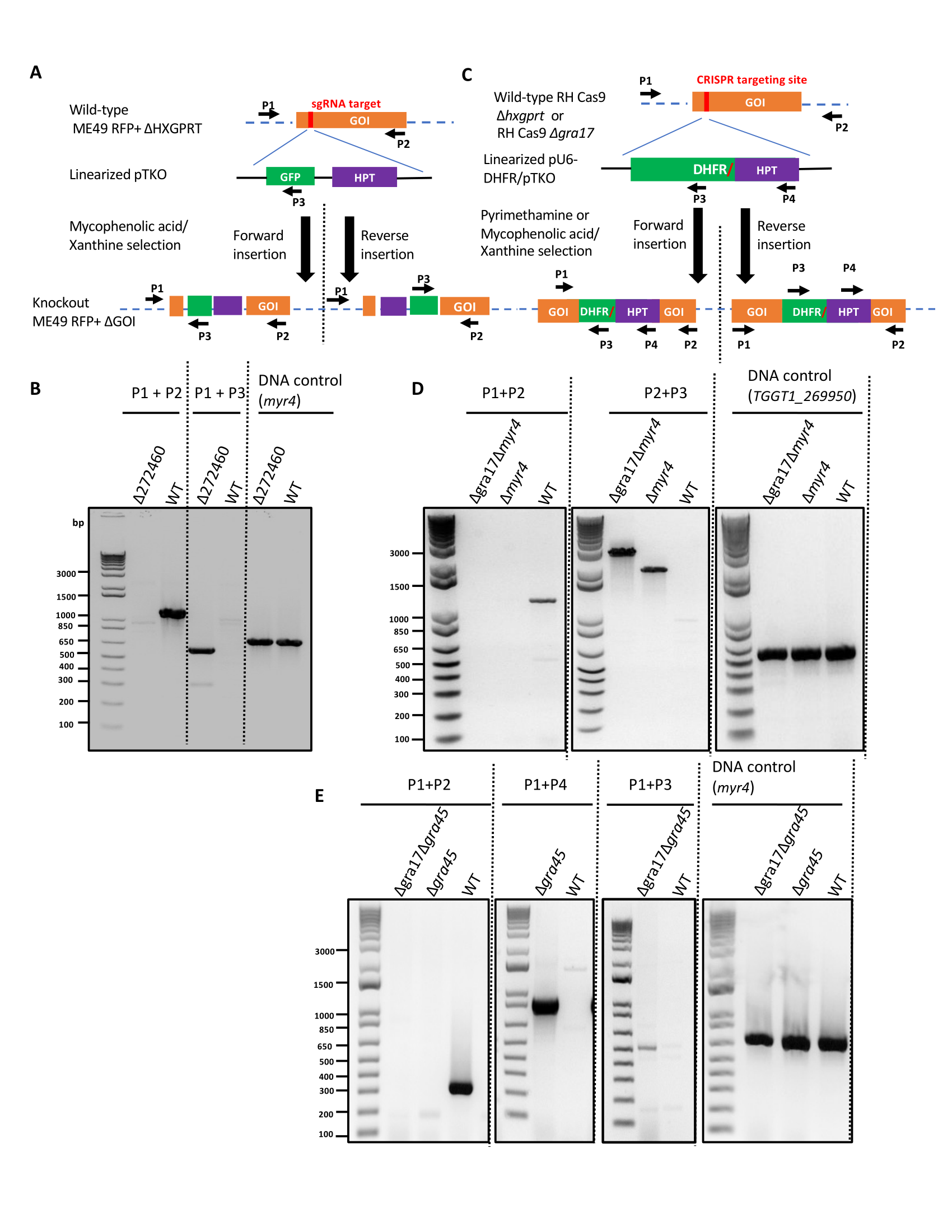

Supplement: S6 Fig — Shown in A) is the schematic diagram of the strategy used to delete GRA72 in the type 2 strain and indicated by a red box is the CRISPR/Cas9-targeting site. Linearized pTKO plasmid carrying a GFP and an HXGPRT selection cassette was used as a repair template and the selection was performed with mycophenolic acid and xanthine. B) Disruption of the gene of interest (GOI) was confirmed with primers P1 and P2 amplifying a region in the GOI while MYR4 was used as a PCR control. Insertion of a repair template was confirmed with primers P1+P3. C) Shown is the schematic diagram of the strategy used to delete a GOI in RH-Cas9 Δhxgprt or RH-Cas9 Δgra17 parasites using either a DHFR or HXGPRT resistance cassette. D) Disruption of the GOI was confirmed with primers P1 and P2. Insertion of a repair template was confirmed by primers P1+P3/P4 or P2+P3. (TIF) [file ppat.1011543.s010.tif]

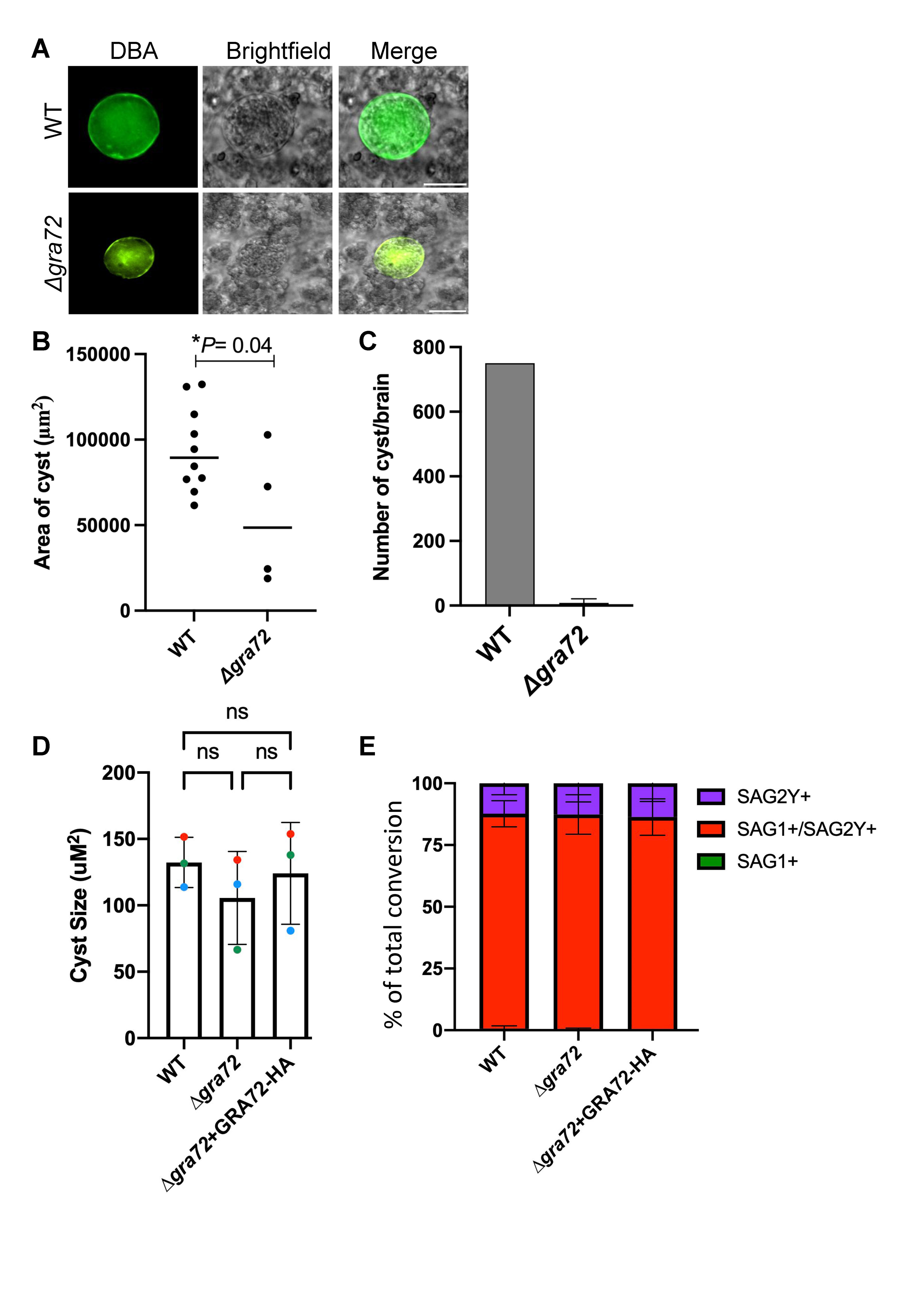

Supplement: S7 Fig — Surviving CD-1 mice from Fig 7 were sacrificed and the number and size of cysts was quantified. A) Representative images of wild-type or Δgra72 cysts. Scale bars indicate 10 μm. B) Cyst area was measured from wild-type (n = 10 cysts) and Δgra72 (n = 4 cysts) cysts. Statistical significance was determined by unpaired t-test and error bars indicate SD. C) Number of cyst/brain 30 days post infection, brains were isolated from wild type (n = 1) or Δgra72 (n = 8) infected mice and cyst numbers were quantified. D) Indicated parasites (in the ME49 type II strain) were converted to in vitro cysts and after 14 days cells were fixed and the cyst wall was stained with Dolichos biflorus agglutinin. Cyst size was quantified from at least 30 cysts per experiment. Shown are the average cyst sizes for 3 independent experiments. One-way ANOVA with Tukey’s multiple comparison test was used to determine significance (ns = not significant). E) Parasites were converted as in D but after fixation parasites were stained with antibodies against the bradyzoite SAG2Y and tachyzoite SAG1 surface markers. The percentage of vacuoles with at least 4 or more parasites that stained for the indicated surface markers is indicated. Shown are averages and SD from 3 independent experiments. (TIF) [file ppat.1011543.s011.tif]
